# Development of allogeneic HSC-engineered iNKT cells for off-the-shelf cancer immunotherapy

**DOI:** 10.1016/j.xcrm.2021.100449

**Published:** 2021-11-16

**Authors:** Yan-Ruide Li, Yang Zhou, Yu Jeong Kim, Yanni Zhu, Feiyang Ma, Jiaji Yu, Yu-Chen Wang, Xianhui Chen, Zhe Li, Samuel Zeng, Xi Wang, Derek Lee, Josh Ku, Tasha Tsao, Christian Hardoy, Jie Huang, Donghui Cheng, Amélie Montel-Hagen, Christopher S. Seet, Gay M. Crooks, Sarah M. Larson, Joshua P. Sasine, Xiaoyan Wang, Matteo Pellegrini, Antoni Ribas, Donald B. Kohn, Owen Witte, Pin Wang, Lili Yang

**Affiliations:** 1 Department of Microbiology, Immunology & Molecular Genetics, University of California, Los Angeles, Los Angeles, CA 90095, USA; 2 Department of Molecular, Cell and Developmental Biology, College of Letters and Sciences, University of California, Los Angeles, Los Angeles, CA 90095, USA; 3 Department of Pharmacology and Pharmaceutical Sciences, University of Southern California, Los Angeles, CA 90089, USA; 4 Eli and Edythe Broad Center of Regenerative Medicine and Stem Cell Research, University of California, Los Angeles, Los Angeles, CA 90095, USA; 5 Department of Medicine, University of California, Los Angeles, Los Angeles, CA 90095, USA; 6 Department of Pathology and Laboratory Medicine, University of California, Los Angeles, Los Angeles, CA 90095, USA; 7 Jonsson Comprehensive Cancer Center, David Geffen School of Medicine, University of California, Los Angeles, Los Angeles, CA 90095, USA; 8 Department of Pediatrics, University of California, Los Angeles, Los Angeles, CA 90095, USA; 9 Department of Internal Medicine, University of California, Los Angeles, Los Angeles, CA 90095, USA; 10 Division of Hematology/Oncology, Department of Pediatrics, David Geffen School of Medicine, University of California, Los Angeles, Los Angeles, CA 90095, USA; 11 Department of Molecular and Medical Pharmacology, University of California, Los Angeles, Los Angeles, CA 90095, USA; 12 Parker Institute for Cancer Immunotherapy, University of California, Los Angeles, Los Angeles, CA 90095, USA; 13 Molecular Biology Institute, University of California, Los Angeles, Los Angeles, CA 90095, USA

**Keywords:** hematopoietic stem cell, invariant natural killer T cells, cancer immunotherapy, allogeneic off-the-shelf cell therapy, chimeric antigen receptor, allogeneic HSC-engineered iNKT cells, HLA-ablated universal HSC-iNKT cells, CAR-engineered conventional αβ T cells, graft-versus-host disease, allorejection

## Abstract

Cell-based immunotherapy has become the new-generation cancer medicine, and “off-the-shelf” cell products that can be manufactured at large scale and distributed readily to treat patients are necessary. Invariant natural killer T (iNKT) cells are ideal cell carriers for developing allogeneic cell therapy because they are powerful immune cells targeting cancers without graft-versus-host disease (GvHD) risk. However, healthy donor blood contains extremely low numbers of endogenous iNKT cells. Here, by combining hematopoietic stem cell (HSC) gene engineering and *in vitro* differentiation, we generate human allogeneic HSC-engineered iNKT (^Allo^HSC-iNKT) cells at high yield and purity; these cells closely resemble endogenous iNKT cells, effectively target tumor cells using multiple mechanisms, and exhibit high safety and low immunogenicity. These cells can be further engineered with chimeric antigen receptor (CAR) to enhance tumor targeting or/and gene edited to ablate surface human leukocyte antigen (HLA) molecules and further reduce immunogenicity. Collectively, these preclinical studies demonstrate the feasibility and cancer therapy potential of ^Allo^HSC-iNKT cell products and lay a foundation for their translational and clinical development.

## Highlights

• Allogeneic HSC-iNKT cells are generated *in vitro* at high yield and purity
• Allogeneic HSC-iNKT cells effectively target tumor cells using multiple mechanisms
• Allogeneic HSC-iNKT cells exhibit high safety and low immunogenicity
• A preclinical study demonstrated feasibility, safety, and cancer therapy potential

## Graphical abstract

**Figure:**
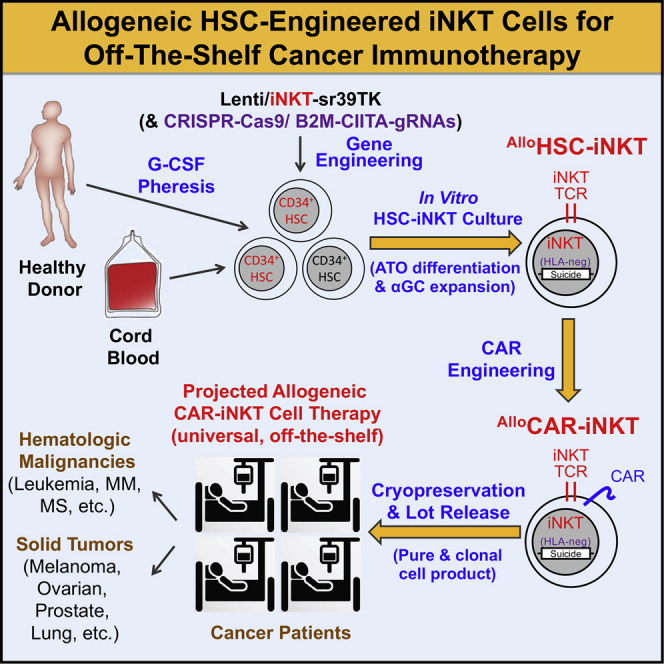

## Teaser

Li et al. report the preclinical development of allogeneic-hematopoietic-stem-cell-engineered invariant natural killer T (HSC-iNKT) cells for off-the-shelf cancer therapy. Allogeneic HSC-iNKT cells demonstrate cancer therapy potential and a high safety profile.

## Introduction

Over the past decade, immunotherapy has become the new-generation cancer medicine.^1^, ^2^, ^3^ In particular, T-cell-based cancer therapy has shown great promise.^4^ An outstanding example is the chimeric antigen receptor (CAR)-engineered T (CAR-T) cell adoptive therapy, which targets certain blood cancers at impressive efficacy and has been approved by the US Food and Drug Administration (FDA) to treat CD19^+^ B cell malignancies.^4^, ^5^, ^6^ Adoptive transfer of *in vitro* expanded tumor-infiltrating T lymphocytes (TILs) and T cell receptor (TCR)-engineered T cells also show promise in treating some blood cancers and solid tumors in the clinic.^7^^,^^8^ However, most of the current T cell therapies fall in the category of autologous cell therapy, wherein T cells collected from a patient are manufactured and used to treat that single patient. Such an approach is time consuming, logistically challenging, and costly; furthermore, for patients with heavily lymphopenic pretreatment or rapidly proliferative disease, it might not always be possible to produce autologous cell products.^5^^,^^9^ Allogenic cell products that can be manufactured at large scale and distributed readily to treat a broad range of cancer patients therefore are in great demand.

Conventional αβ T cells have been utilized for generating allogeneic cell products; however, these T cells risk inducing graft-versus-host disease (GvHD) in allogeneic hosts due to histocompatibility leukocyte antigen (HLA) incompatibility, thereby requiring additional gene editing to ablate their endogenous TCR expression that may potentially increase manufacture complexity.^10^, ^11^, ^12^, ^13^ Innate immune cells such as natural killer (NK) cells that have no GvHD risk have been investigated; however, NK cells may have limited *in vivo* clonal expansion and antitumor performance compared to T cells.^14^^,^^15^

Invariant NK T (iNKT) cells are a small population of αβ T lymphocytes.^16^ iNKT cells have several unique features, making them ideal for developing off-the-shelf cellular therapy for cancer. Compared to conventional T cells, iNKT cells can attack tumor cells using multiple mechanisms and at higher efficacy; can more effectively traffic to and infiltrate solid tumors; can alter solid tumor immunosuppressive microenvironment; and, most importantly, do not induce GvHD.^17^, ^18^, ^19^, ^20^, ^21^, ^22^, ^23^, ^24^, ^25^ However, human blood contains extremely low numbers of iNKT cells (0.001%–1%), making it very difficult to reliably grow large numbers of allogeneic iNKT cells for cell therapies.^26^ Moreover, allogeneic iNKT products expanded from blood may contain bystander allogeneic conventional αβ T cells and thus risk inducing GvHD. Technology breakthroughs are needed to exploit the allogeneic cell therapy potential of iNKT cells.

Previously, we have established a method to generate large numbers of iNKT cells through TCR gene engineering of hematopoietic stem cells (HSCs) followed by *in vivo* reconstitution; using this method, we have successfully generated both mouse and human HSC-engineered iNKT (HSC-iNKT) cells.^27^^,^^28^ However, such an *in vivo* approach cannot be used to produce off-the-shelf mature allogeneic iNKT cells.^27^^,^^28^ Here, we intended to build on the HSC-iNKT engineering approach and develop an *in vitro* culture method to produce large numbers of off-the-shelf human iNKT cells for allogeneic cell therapy applications. We report the preclinical development of the proposed allogeneic HSC-iNKT cell therapy, demonstrating its manufacture feasibility, cancer therapy potential, and high safety profile.

## Results

### Generation of allogeneic HSC-engineered iNKT (^Allo^HSC-iNKT) cells

**Figure 1:**
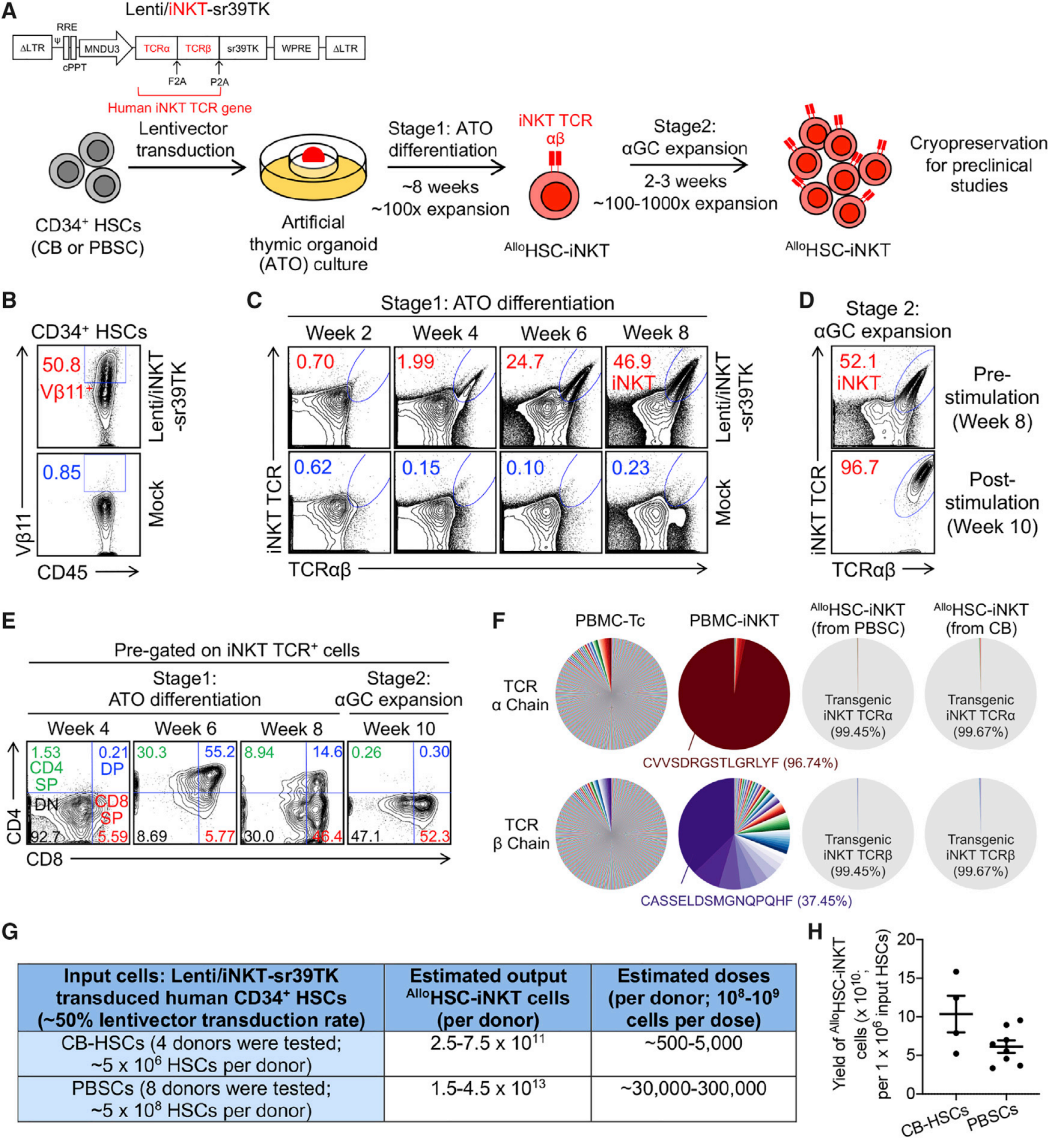
*In vitro* generation of allogenic HSC-engineered iNKT (^Allo^HSC-iNKT) cells (A) Experimental design to generate ^Allo^HSC-iNKT cells *in vitro*. HSC, hematopoietic stem cell; CB, cord blood; PBSC, peripheral blood stem cell; αGC, α-galactosylceramide; Lenti/iNKT-sr39TK, lentiviral vector encoding an iNKT TCR gene and an sr39TK suicide/positron emission tomography (PET) imaging gene. (B–E) Fluorescence-activated cell sorting (FACS) monitoring of ^Allo^HSC-iNKT cell generation. (B) Intracellular expression of iNKT TCR (identified as Vβ11^+^) in CD34^+^ HSCs at 72 h after lentivector transduction. (C) Generation of iNKT cells (identified as iNKT TCR^+^TCRαβ^+^ cells) during stage 1 ATO differentiation culture. A 6B11 monoclonal antibody was used to stain iNKT TCR. (D) Expansion of iNKT cells during stage 2 αGC expansion culture. (E) Expression of CD4/CD8 co-receptors on ^Allo^HSC-iNKT cells during stage 1 and stage 2 cultures. DN, CD4/CD8 double negative; CD4 SP, CD4 single positive; DP, CD4/CD8 double positive; CD8 SP, CD8 single positive. (F) Single-cell TCR sequencing analysis of ^Allo^HSC-iNKT cells. Healthy donor peripheral blood mononuclear cell (PBMC)-derived conventional αβ T (PBMC-Tc) and iNKT (PBMC-iNKT) cells were included as controls. The relative abundance of each unique T cell receptor (TCR) sequence among the total unique sequences identified for individual cells is represented by a pie slice. (G) Table summarizing experiments that have successfully generated ^Allo^HSC-iNKT cells. (H) Yields of ^Allo^HSC-iNKT cells generated from multiple HSC donors. Representative of 1 (F) and >10 experiments (A–E).

Human CD34^+^ cells collected from either cord blood (CB) or granulocyte-colony-stimulating factor (G-CSF)-mobilized human peripheral blood stem cells (PBSCs) were transduced with a Lenti/iNKT-sr39TK vector and then cultured *in vitro* in a two-stage artificial thymic organoid (ATO)/α-galactosylceramide (αGC) culture system (Figure 1A). Note CD34^+^ cells comprise both hematopoietic stem and progenitor cells; in this report we refer to CD34^+^ cells as HSCs. The Lenti/iNKT-sr39TK vector has been previously used to develop an autologous HSC-engineered iNKT cell therapy for cancer^28^; ATO is an *in vitro* 3D culture that supports the human HSC differentiation into T cells,^29^^,^^30^ while αGC is a synthetic agonist glycolipid ligand that specifically stimulates iNKT cells.^16^ We routinely achieved over 50% lentivector transduction rate of HSCs (Figure 1B). Transduced HSCs were then placed in the stage 1 ATO culture, where they differentiated into human iNKT cells over a course of 8 weeks with over 100-fold expansion (Figures 1A and 1C). At the end of stage 1 culture, ATOs were dissociated into single cells that were then placed in the stage 2 αGC expansion culture for another 2–3 weeks, resulting in another 100- to 1,000-fold expansion and an ^Allo^HSC-iNKT cell product of high yield and purity (Figures 1A and 1D).

In the ATO/αGC culture, ^Allo^HSC-iNKT cells followed a typical iNKT cell development path defined by CD4/CD8 co-receptor expression.^31^ During stage 1, ^Allo^HSC-iNKT cells transited from CD4^−^CD8^−^ (DN) to CD4^+^CD8^+^ (DP), then toward CD4^−^CD8^+/−^ (Figure 1E). At the end of stage 2, the majority (>99%) of ^Allo^HSC-iNKT cells showed a CD4^-^CD8^+/−^ (CD8 SP/DN) phenotype (Figure 1E). Note that the end ^Allo^HSC-iNKT cell product did not contain a CD4^+^CD8^−^ (CD4 SP) population that are present in the endogenous human iNKT cells (Figure 1E).^31^ In general, CD8 SP/DN human iNKT cells are considered to be proinflammatory and highly cytotoxic and thereby are desirable for cancer immunotherapy.^24^^,^^31^, ^32^, ^33^, ^34^, ^35^

Flow cytometry analysis showed that the ^Allo^HSC-iNKT cell product comprised high-purity transgenic iNKT cells (Figure 1D); single-cell TCR sequencing analysis confirmed that these ^Allo^HSC-iNKT cells uniformly expressed the transgenic iNKT TCRs while nearly undetectable randomly recombined endogenous αβ TCRs (Figure 1F). In sharp contrast to ^Allo^HSC-iNKT cells, conventional αβ T cells isolated from health donor periphery blood (denoted as PBMC-Tc cells) expressed highly diverse endogenously recombined αβ TCRs, whereas iNKT cells isolated from health donor periphery blood (denoted as PBMC-iNKT cells) expressed a conserved invariant TCR α chain (Vα24-Jα18) and limited diverse TCR β chains (dominantly Vβ11) (Figure 1F).

The manufacture of ^Allo^HSC-iNKT cells was highly robust; we generated ^Allo^HSC-iNKT cell products of high purity and yield from all 12 donors tested (4 CB-HSCs and 8 PBSCs) (Figures 1G and 1H). Based on our results, it was estimated that from one CB donor (comprising ∼5 × 10^6^ CB-HSCs), ∼5 × 10^11 Allo^HSC-iNKT cells could be generated that can potentially be formulated into ∼500–5,000 doses (∼10^8^-10^9^ cells per dose based on the approved CAR-T cell therapy doses)^4^; from one PBSC donor (comprising ∼5 × 10^8^ PBSCs), ∼3 × 10^13 Allo^HSC-iNKT cells could be generated that can potentially be formulated into ∼30,000–300,000 doses (Figure 1G). The resulting ^Allo^HSC-iNKT cell product contained pure transgenic iNKT cells and nearly undetectable bystander conventional αβ T cells, thereby lack of GvHD risk and suitable for “off-the-shelf” application.

### Phenotype and functionality of ^Allo^HSC-iNKT cells

**Figure 2:**
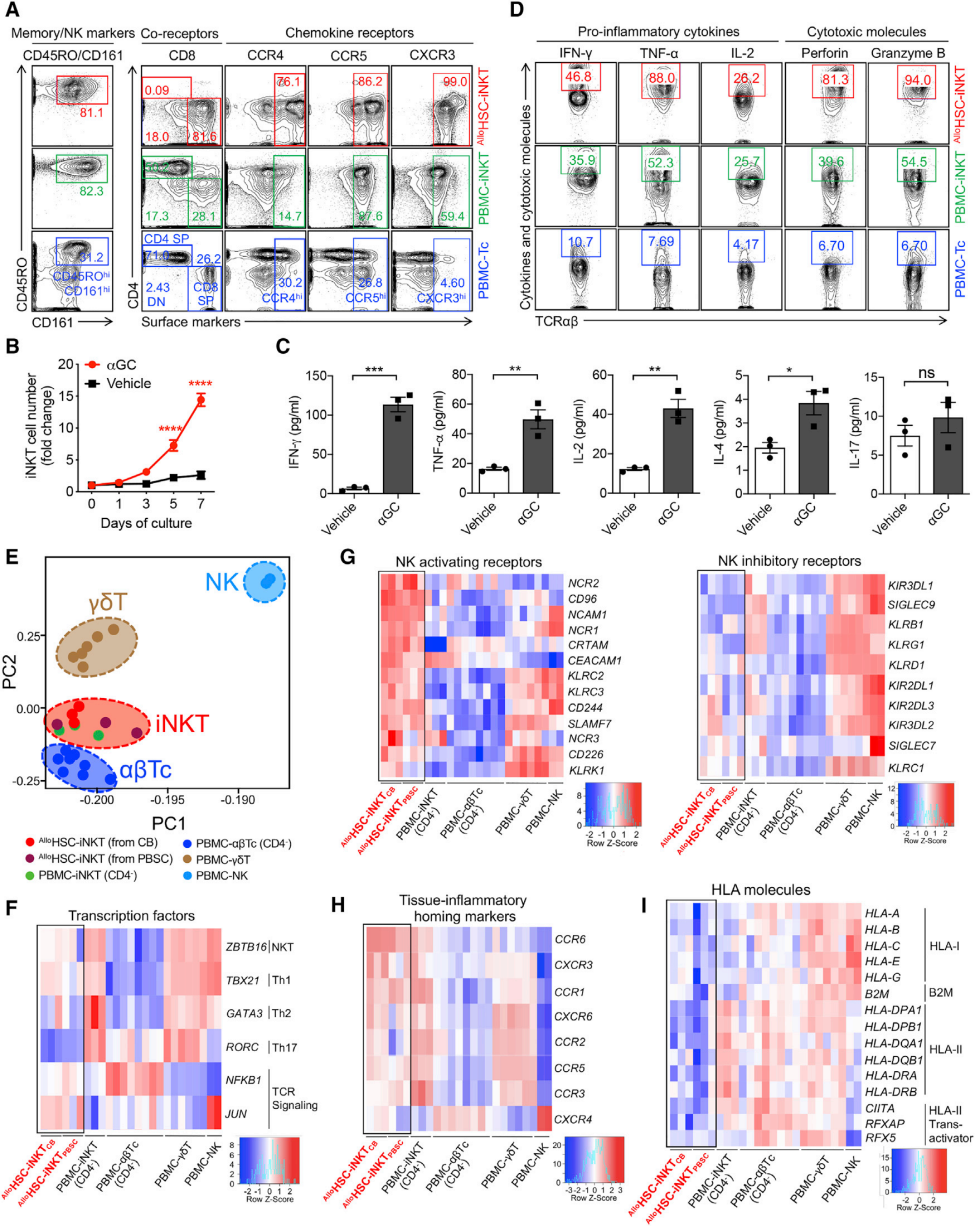
Characterization and gene profiling of ^Allo^HSC-iNKT cells (A) FACS detection of surface markers on ^Allo^HSC-iNKT cells. PBMC-iNKT and PBMC-Tc cells were included as controls. (B and C) Antigen responses of ^Allo^HSC-iNKT cells. ^Allo^HSC-iNKT cells were cultured for 7 days, in the presence or absence of αGC (denoted as αGC or Vehicle, respectively). (B) Cell growth curve (n = 3). (C) ELISA analyses of cytokine (IFN-γ, TNF-α, IL-2, IL-4 and IL-17) production at day 7 post αGC stimulation (n = 3). (D) FACS detection of intracellular cytokines and cytotoxic molecules in ^Allo^HSC-iNKT cells. PBMC-iNKT and PBMC-Tc cells were included as controls. (E–I) Deep RNA-seq analysis of ^Allo^HSC-iNKT cells generated from CB- or PBSC-derived CD34^+^ HSCs (n = 3 for each). Healthy donor PBMC-derived conventional CD4^−^ αβ T (PBMC-αβTc; n = 8), CD4^−^ iNKT (PBMC-iNKT; n = 3), γδ T (PBMC- γδT; n = 6), and NK (PBMC-NK; n = 2) cells were included as controls. N indicates different donors. (E) Principal-component analysis (PCA) plot showing the ordination of all six cell types. (F–I) Heatmaps showing the expression of selected genes encoding transcription factors (F), NK activating and inhibitory receptors (G), tissue inflammatory homing markers (H), and HLA molecules (I) for all six cell types. Representative of 1 (E–I) and 3 (A–D) experiments. Data are presented as the mean ± SEM ns, not significant, ∗p < 0.05, ∗∗p < 0.01, ∗∗∗p < 0.001, ∗∗∗∗p < 0.0001, by Student’s t test.

Next, we analyzed the phenotype and functionality of ^Allo^HSC-iNKT cells in comparison with endogenous human PBMC-iNKT and PBMC-Tc cells. ^Allo^HSC-iNKT cells displayed a phenotype closely resembling PBMC-iNKT cells but distinct from PBMC-Tc cells; they expressed high levels of memory T cell markers (i.e., CD45RO), NK cell markers (i.e., CD161), and peripheral tissue and inflammatory site homing markers (i.e., CCR4, CCR5 and CXCR3) (Figure 2A). When stimulated with αGC, ^Allo^HSC-iNKT cells proliferated vigorously (Figure 2B) and secreted high levels of Th0/Th1 cytokines (i.e., interferon-γ [IFN-γ], tumor necrosis factor α [TNF-α], and interleukin-2 [IL-2]) but limited amounts of Th2 cytokines (i.e., IL-4) and Th17 cytokines (i.e., IL-17) (Figure 2C), indicating a Th0/Th1-prone functionality of ^Allo^HSC-iNKT cells that agrees with their CD8 SP/DN phenotype (Figures 1E and 2A).^24^^,^^31^, ^32^, ^33^ Intracellular staining showed that at the single-cell level, ^Allo^HSC-iNKT cells produced exceedingly high levels of effector cytokines (i.e., IFN-γ, TNF-α, and IL-2) and cytotoxic molecules (i.e., perforin and granzyme B) (Figure 2D). The ability to generate excess amounts of antitumor effector molecules is a promising signature of iNKT cells for cancer immunotherapy.^26^^,^^36^

### Transcriptome profiling of ^Allo^HSC-iNKT cells

To fully characterize ^Allo^HSC-iNKT cells, we performed deep RNA sequencing (RNA-seq) analysis of these cells; ^Allo^HSC-iNKT cells generated from both CB and PBSC CD34^+^ HSCs were studied. Healthy donor PBMC-derived endogenous iNKT (PBMC-iNKT), conventional αβ T (PBMC-αβTc), γδ T (PBMC-γδT), and NK (PBMC-NK) cells were included as controls. All cell types were prepared from multiple donors. Because ^Allo^HSC-iNKT cells were dominantly CD4^−^ (CD8 SP/DN), the CD4^−^ subpopulations of PBMC-iNKT (CD8 SP/DN) and PBMC-αβTc (CD8^+^) cells were analyzed in this experiment.

Principal-component analysis (PCA) of the global gene expression profiles showed that ^Allo^HSC-iNKT cells (both CB and PBSC-derived) were located the closest to PBMC-iNKT cells, next to PBMC-αβTc and PBMC-γδT cells, and the furthest from PBMC-NK cells, validating the iNKT cell nature of ^Allo^HSC-iNKT cells (Figure 2E).^37^

“Master” transcription factor gene profiling analysis revealed a distinctive signature of ^Allo^HSC-iNKT cells; they expressed high levels of *ZBTB16* that encodes PLZF, a signature transcription factor of innate T (e.g., iNKT and γδT) cells and NK cells^38^; they expressed high levels of *TBX21* that encodes T-bet, an essential transcription factor regulating Th1 polarization of T cells^39^; they expressed low levels of *GATA3* and *RORC* that respectively encode GATA3 and RORγ, critical transcription factors regulating Th2 and Th17 polarization of T cells^40^; and they expressed high levels of *NFKB1* and *JUN* that respectively encode NF-κB1 and c-Jun, important transcription factors for TCR signaling (Figure 2F).^41^^,^^42^ These transcription factors have been indicated to play important roles in regulating iNKT cell development and functionality.^43^, ^44^, ^45^ Of note, the *TBX21*^high^*GATA3*^low^*RORC*^low^ expression profile of ^Allo^HSC-iNKT cells consists with their Th0/Th1-prone cytokine production profile (Figure 2C).

Further analysis of the various genes related to antitumor effector functions (e.g., genes encoding activation/homing markers, cytokines, and cytotoxic molecules) revealed ^Allo^HSC-iNKT cells to be highly potent effector cells, in agreement with their *in vitro* phenotype and functionality characterization (Figures 2A–2D). In addition, several interesting gene signatures stood out, which are highlighted below.

Typical iNKT cells exert NK function besides T cell function, via surface expression of NK receptors.^16^^,^^18^^,^^46^ Interestingly, compared to PBMC-iNKT and even PBMC-NK cells, ^Allo^HSC-iNKT cells expressed exceedingly high levels of NK activating receptor genes (e.g., *NCAM1*, *NCR1*, *NCR2*, *KLR2*, and *KLR3*) but low levels of NK inhibitory receptor genes (e.g., *KIR3DL1*, *KIR3DL2*, *KIR2DL1*, and *KIR2DL2*), suggesting that ^Allo^HSC-iNKT cells might exhibit a superior antitumor NK function (Figure 2G).

Tissue inflammatory homing markers expressed on effector immune cells enable them to access inflammatory tissues including tumor sites.^32^^,^^47^ ^Allo^HSC-iNKT cells expressed exceedingly high levels of multiple tissue inflammatory homing marker genes (e.g., *CCR1*, *CCR2*, *CCR3*, *CCR5*, *CCR6*, and *CXCR3*), comparable to those of endogenous innate T cells (i.e., PBMC-iNKT and PBMC-γδT cells) but significantly higher than those of endogenous conventional αβ T and NK cells (i.e., PBMC-αβTc and PBMC-NK cells), suggesting a strong capacity of ^Allo^HSC-iNKT cells to home to and penetrate tumor sites (Figure 2H).

HLA incompatibility may trigger host T-cell-mediated allorejection of adoptively transferred allogeneic cellular products, thereby limiting their therapeutic efficacy.^48^^,^^49^ Interestingly, compared to all of the endogenous T cells (i.e., PBMC-iNKT, PBMC-αβTc, and PBMC-γδT cells) and NK cells (i.e., PBMC-NK cells) tested, ^Allo^HSC-iNKT cells expressed much lower levels of HLA-expression-related genes (e.g., genes encoding HLA-I molecules, B2M, HLA-II molecules, and HLA-II transactivators), suggesting that ^Allo^HSC-iNKT cells might naturally resist allorejection and thereby have certain advantages over many PBMC-derived allogeneic cell products for off-the-shelf cell therapy (Figure 2I).

### Tumor targeting of ^Allo^HSC-iNKT cells through intrinsic NK function

**Figure 3:**
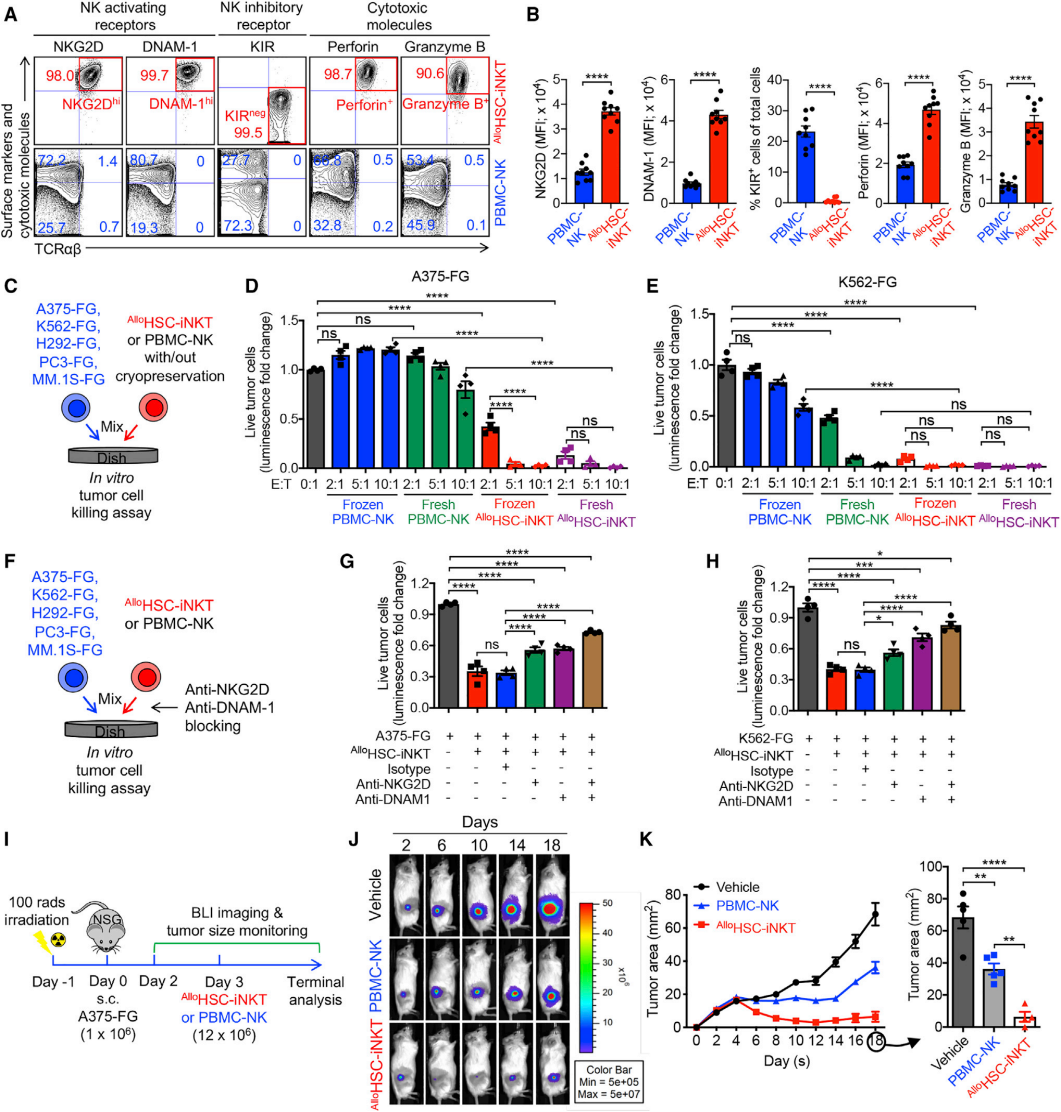
Tumor targeting of ^Allo^HSC-iNKT cells through intrinsic NK function (A and B) FACS analyses of surface NK receptor expression and intracellular cytotoxic molecule production by ^Allo^HSC-iNKT cells. PBMC-NK cells were included as a control. (A) Representative FACS plots. (B) Quantification of (A) (n = 9). (C–E) *In vitro* direct killing of human tumor cells by ^Allo^HSC-iNKT cells. PBMC-NK cells were included as a control. Both fresh and frozen-thawed cells were studied. Five human tumor cell lines were studied: A375 (melanoma), K562 (myelogenous leukemia), H292 (lung cancer), PC3 (prostate cancer), and MM.1S (multiple myeloma). All tumor cell lines were engineered to express firefly luciferase and green fluorescence protein (FG) dual reporters. (C) Experimental design. (D and E) Tumor killing data of A375-FG human melanoma cells (D) and K562-FG human myelogenous leukemia cells (E) at 24 h (n = 4). (F–H) Tumor killing mechanisms of ^Allo^HSC-iNKT cells. NKG2D- and DNAM-1-mediated pathways were studied. (F) Experimental design. (G) Tumor killing data of A375-FG human melanoma cells at 24 h (tumor/iNKT ratio 1:2; n = 4). (H) Tumor killing data of K562-FG human myelogenous leukemia cells at 24 h (tumor/iNKT ratio 1:1; n = 4). (I–K) Studying the *in vivo* antitumor efficacy of ^Allo^HSC-iNKT cells in an A375-FG human melanoma xenograft NSG mouse model. (I) Experimental design. BLI, live animal bioluminescence imaging. (J) BLI images showing tumor loads in experimental mice over time. (K) Tumor size measurements over time (n = 4–5). Representative of three experiments. Data are presented as the mean ± SEM. ns, not significant; ∗p < 0.05; ∗∗p < 0.01; ∗∗∗p < 0.001; ∗∗∗∗p < 0.0001 by Student’s t test (B) or one-way ANOVA (D, E, G, H, and K). See also Figure S1.

Following the NK lead of our RNA-seq study (Figure 2G), we investigated the NK phenotype and antitumor function of ^Allo^HSC-iNKT cells in comparison with those of endogenous PBMC-NK cells. Flow cytometry analysis of cell surface markers showed that ^Allo^HSC-iNKT cells expressed significantly higher levels of NK activating receptors (i.e., NKG2D and DNAM-1) while nearly undetectable NK inhibitory receptors (i.e., killer cell immunoglobulin-like receptors, KIRs) (Figures 3A and 3B). NK activating receptors recognize stress molecules (e.g., MIC-A/B and UL16-binding protein 1-4, ULBP1-4, recognized by NKG2D and CD112 and CD155 recognized by DNAM-1) upregulated on many tumor cells and trigger tumor targeting,^50^, ^51^, ^52^ while NK inhibitory receptors recognize matched “self” major histocompatibility complex (MHC) molecules and suppress tumor killing.^53^^,^^54^ Flow cytometry analysis of intracellular effector molecules showed that compared to PBMC-NK cells, ^Allo^HSC-iNKT cells produced exceedingly higher levels of cytotoxic molecules (i.e., perforin and granzyme B) (Figures 3A and 3B). Collectively, these results confirmed a promising antitumor potential of ^Allo^HSC-iNKT cells through their intrinsic NK function.

An attractive feature of NK-cell-based cancer immunotherapy is its capacity to target a broad range of tumor cells independent of HLA and tumor antigen restrictions.^50^ However, NK-cell-based therapy also confronts several significant challenges, such as the limited *in vivo* efficacy of NK cells, as well as their intolerance to cryopreservation, that pose a significant technical hurdle to their clinical and commercial applications.^55^^,^^56^ Based on the “super-active” NK phenotype of ^Allo^HSC-iNKT cells, we wondered whether these cells might exhibit improved antitumor NK function. Moreover, unlike NK cells, iNKT cells can resist cryopreservation^57^; we therefore wondered whether ^Allo^HSC-iNKT cells might also improve on this aspect.

Using an *in vitro* tumor cell killing assay, we evaluated the tumor killing efficacy of ^Allo^HSC-iNKT cells in comparison with PBMC-NK cells (Figure 3C). Five human tumor cell lines were used as targets, including a leukemia cell line (K562), a melanoma cell line (A375), a lung cancer cell line (H292), a prostate cancer cell line (PC3), and a multiple myeloma cell line (MM.1S). All five tumor cell lines were engineered to overexpress the firefly luciferase (Fluc) and enhanced green fluorescence protein (EGFP) dual reporters to enable the convenient monitoring of these tumor cells using either luciferase assay or flow cytometry (Figure S1A). Compared to PBMC-NK cells, ^Allo^HSC-iNKT cells exhibited a significantly enhanced tumor killing efficacy across all five tumor cell lines (Figures 3D, 3E, and S1B–S1D). Interestingly, ^Allo^HSC-iNKT cells sustained strong tumor killing efficacy after cryopreservation, whereas PBMC-NK cells were sensitive to freeze-thaw cycles and showed greatly reduced viability and antitumor capacity following cryopreservation (Figures 3D, 3E, and S1B–S1D). Blocking of NK activating receptors (i.e., NKG2D and DNAM-1) reduced tumor killing efficacy of ^Allo^HSC-iNKT cells (Figures 3F–3H and S1E–S1G), confirming their NK-activating-receptor-mediated tumor-targeting function.

Next we evaluated the *in vivo* antitumor efficacy of ^Allo^HSC-iNKT cells using a human melanoma xenograft NSG (NOD.Cg-Prkdc^scid^Il2rg^tm1Wjl^/SzJ) mouse model. A375-FG tumor cells were subcutaneously inoculated into NSG mice to form solid tumors, followed by a paratumoral injection of ^Allo^HSC-iNKT or PBMC-NK cells (Figure 3I). ^Allo^HSC-iNKT cells effectively suppressed tumor growth at an efficacy higher than that of PBMC-NK cells, as evidenced by time-course live animal bioluminescence imaging (BLI) monitoring (Figures 3J and S1H), tumor size measurement (Figure 3K), and terminal tumor weight assessment (Figure S1I).

Taken together, these studies support a cancer therapy potential of ^Allo^HSC-iNKT cells through their intrinsic NK function, allowing these cells to target a broad range of tumors independent of HLA and tumor antigen restrictions. Attractively, ^Allo^HSC-iNKT cells may exhibit improved antitumor efficacy and cryopreservation resistance compared to NK-cell-based allogeneic cell therapy products.

### Tumor targeting of ^Allo^HSC-iNKT cells through engineered CARs

CAR-engineered cell therapy has great promise for treating cancer^4^, ^5^, ^6^^,^^58^; we therefore explored the potential of ^Allo^HSC-iNKT cells as the allogeneic cell carriers for CAR-directed off-the-shelf cell therapy. A second-generation B cell maturation antigen (BCMA)-targeting CAR (BCAR) was used for this study (Figure S2A); this BCAR contains 4-1BB and CD3ζ signaling domains and has shown clinical efficacy in treating human multiple myeloma (MM).^59^

**Figure 4:**
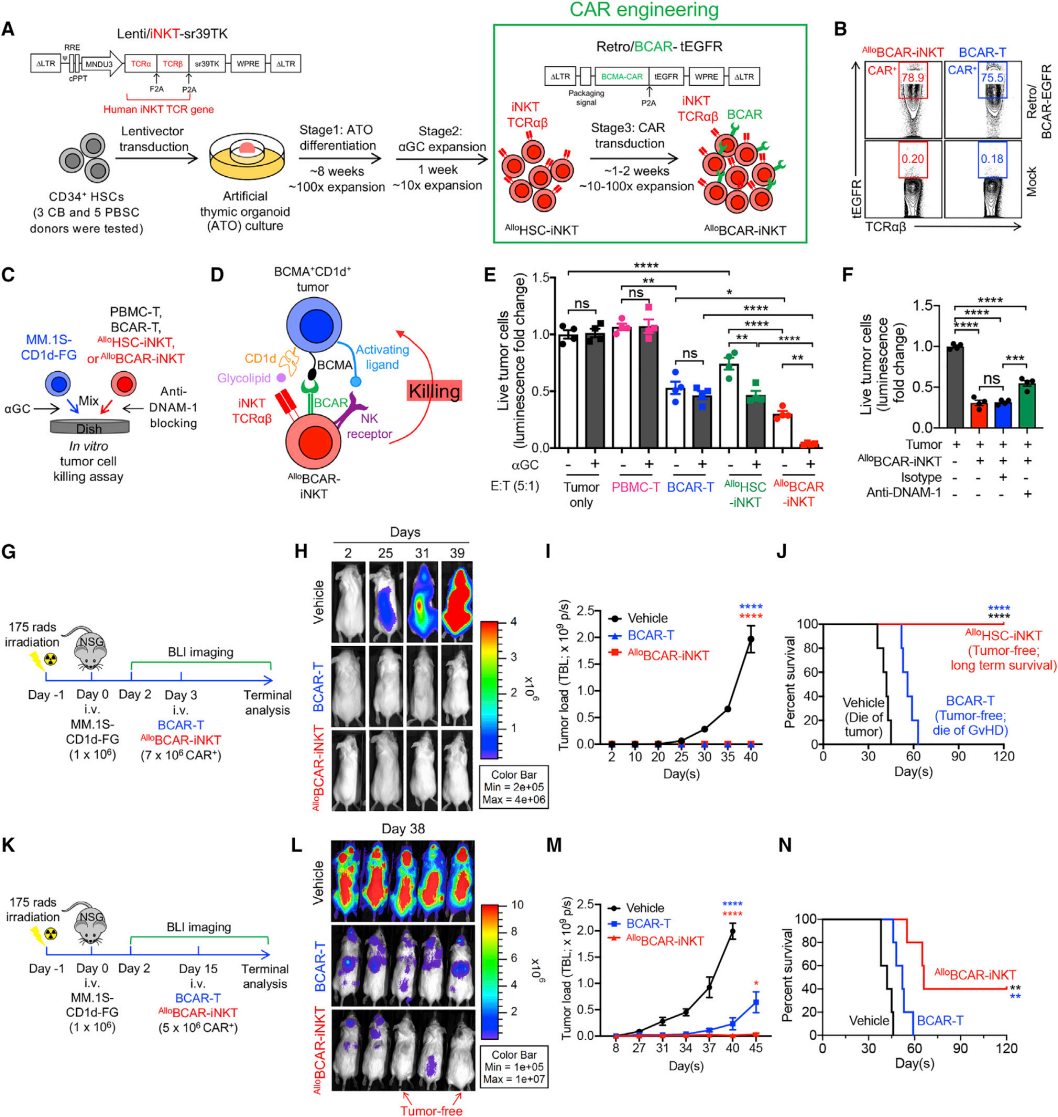
Tumor targeting of ^Allo^HSC-iNKT cells through engineered chimeric antigen receptors (CARs) (A) Experimental design to generate BCMA CAR-engineered ^Allo^HSC-iNKT (^Allo^BCAR-iNKT) cells *in vitro*. BCMA, B cell maturation antigen; BCAR, BCMA CAR; Retro/BCAR-tEGFR, retroviral vector encoding a BCMA CAR gene as well as a truncated epidermal growth factor receptor (tEGFR) reporter gene. tEGFR was used as a staining marker indicating BCAR expression. (B) FACS analysis of BCAR expression (identified as tEGFR^+^) on ^Allo^BCAR-iNKT at 72 h after retrovector transduction. Healthy donor PBMC-T cells transduced with the same Retro/BCAR-tEGFR vector (denoted as BCAR-T cells) were included as a staining control. (C–F) *In vitro* killing of human multiple myeloma cells by ^Allo^BCAR-iNKT cells. MM.1S-CD1d-FG, human MM.1S cell line engineered to overexpress human CD1d as well as FG dual reporters. PBMC-T, BCAR-T, and ^Allo^HSC-iNKT cells were included as effector cell controls. (C) Experimental design. (D) Diagram showing the tumor-targeting triple mechanisms of ^Allo^BCAR-iNKT cells, mediated by NK activating receptors, iNKT TCR, and BCAR. (E) Tumor cell killing by the indicated effector cells with/out the addition of αGC (n = 4). (F) Tumor cell killing by ^Allo^BCAR-iNKT cells with/out the blockade of DNAM-1 (n = 4). Tumor cell killing was analyzed at 8-h after co-culture (effector/tumor ratio 5:1). (G–N) Studying the *in vivo* antitumor efficacy of ^Allo^BCAR-iNKT cells in an MM.1S-CD1d-FG human multiple myeloma xenograft NSG mouse model. Tumor-bearing mice injected with BCAR-T cells or no cells (vehicle) were included as controls. (G–J) Low-tumor-load condition. (G) Experimental design. (H) BLI images showing tumor loads in experimental mice over time. (I) Quantification of (H) (n = 5). (J) Kaplan-Meier survival curves of experimental mice over a period of 4 months after tumor challenge (n = 5). (K–N) High-tumor-load condition. (K) Experimental design. (L) BLI images showing tumor loads in experimental mice at day 38. (M) Quantification of tumor load in experimental mice over time (n = 5). (N) Kaplan-Meier survival curves of experimental mice over a period of 4 months after tumor challenge (n = 5). Representative of two experiments (K–N) and three experiments (A–J). Data are presented as the mean ± SEM. ns, not significant; ∗p < 0.05; ∗∗p < 0.01; ∗∗∗p < 0.001; ∗∗∗∗p < 0.0001 by one-way ANOVA (E, F, I, and M), or log rank (Mantel-Cox) test adjusted for multiple comparisons (J and N). See also Figure S2.

^Allo^HSC-iNKT cells were generated as previously described (Figure 1A); mature ^Allo^HSC-iNKT cells were further transduced with a Retro/BCAR-tEGFR retroviral vector to produce the BCAR-engineered ^Allo^HSC-iNKT cells (denoted as ^Allo^BCAR-iNKT cells) (Figure 4A). The entire culture time (∼10–11 weeks) and cell yield (∼10^11^ per CB donor or ∼10^12^ per PBSC donor) were similar to those of generating non-CAR-engineered ^Allo^HSC-iNKT cells (Figures 1A and 1G). The resulting ^Allo^BCAR-iNKT cells were pure iNKT cells with a high BCAR expression rate (>98% iNKT TCR^+^ and up to 80% BCAR^+^; Figure 4B) and displayed a typical human iNKT cell phenotype and functionality similar to those of ^Allo^HSC-iNKT cells (Figures 2A, 2D, S2C, and S2D). Therefore, ^Allo^HSC-iNKT cells can be effectively engineered to express CARs without compromising cell yield and quality.

To assess the antitumor capacity of ^Allo^BCAR-iNKT cells, we used an established *in vitro* MM.1S-CD1d-FG tumor cell killing assay^28^; non-CAR-engineered ^Allo^HSC-iNKT cells, as well as healthy donor PBMC-derived conventional T cells with or without engineering with the same BCAR (denoted as PBMC-T or BCAR-T cells), were included as controls (Figure 4C). The MM.1S-CD1d-FG cell line was generated by engineering the parental BCMA-positive MM.1S human MM cell line to overexpress human CD1d and the Fluc-EGFP dual reporters,^28^ mimicking a large portion of primary patient MM samples that are BCMA^+^CD1d^+^ (Figure S2B).^60^ This *in vitro* tumor cell killing assay allowed us to evaluate the tumor killing capacity of ^Allo^BCAR-iNKT cells, as well as investigate their possible NK/TCR/CAR triple mechanisms for targeting MM (Figure 4D).

^Allo^HSC-iNKT cells without BCAR engineering were able to kill MM.1S-CD1d-FG tumor cells, while PBMC-T cells could not, indicating an intrinsic antitumor NK function of ^Allo^HSC-iNKT cells (Figures 4E, S1D, and S1G); this intrinsic antitumor NK function was inherited by ^Allo^BCAR-iNKT cells, as confirmed by NK activating receptor (i.e., DNAM-1) blocking assay (Figure 4F). Meanwhile, the tumor cell killing efficacy of ^Allo^HSC-iNKT cells was enhanced by the addition of αGC that did not happen to PBMC-T cells, indicating an iNKT TCR-mediated antitumor function of ^Allo^HSC-iNKT cells; this TCR-mediated antitumor function was also inherited by ^Allo^BCAR-iNKT cells (Figure 4E). Importantly, compared to ^Allo^HSC-iNKT cells, ^Allo^BCAR-iNKT cells showed stronger tumor cell killing, indicating a CAR-mediated antitumor function of ^Allo^BCAR-iNKT cells (Figure 4E). Therefore, ^Allo^BCAR-iNKT cells are able to target MM tumor cells using the NK/TCR/CAR triple mechanisms, which may grant ^Allo^BCAR-iNKT cells a higher tumor-targeting efficacy and an enhanced capacity to counteract tumor antigen escape compared to conventional BCAR-T cells (Figures 4D–4F).^25^^,^^61^^,^^62^

The *in vivo* antitumor efficacy of ^Allo^BCAR-iNKT cells was studied using an established MM.1S-CD1d-FG xenograft NSG mouse model^28^; conventional BCAR-T cells were included as a control. In low-tumor-load conditions, ^Allo^BCAR-iNKT cells eliminated MM tumor cells as effectively as BCAR-T cells (Figures 4H and 4I); however, experimental mice treated with BCAR-T cells eventually died of graft-versus-host disease (GvHD) despite being tumor-free, while experimental mice treated with ^Allo^BCAR-iNKT cells lived long-term with tumor-free and GvHD-free (Figures 4I and 4J). Impressively, in high-tumor-load conditions, ^Allo^BCAR-iNKT cells still managed to suppress tumor growth effectively and achieved tumor clearance in a fraction of experimental mice (two out of five mice); conventional BCAR-T cells suppressed tumor growth less well and could not achieve tumor clearance (Figures 4K–4N and S2E).

Taken together, these studies support an attractive potential of ^Allo^HSC-iNKT cells as off-the-shelf cell carriers for CAR-directed cancer immunotherapy. The high antitumor efficacy and multiple tumor-targeting mechanisms of CAR-engineered ^Allo^HSC-iNKT cells may provide new opportunities to target hard-to-treat tumors and counteract tumor antigen escape.

### Safety study of ^Allo^HSC-iNKT cells

**Figure 5:**
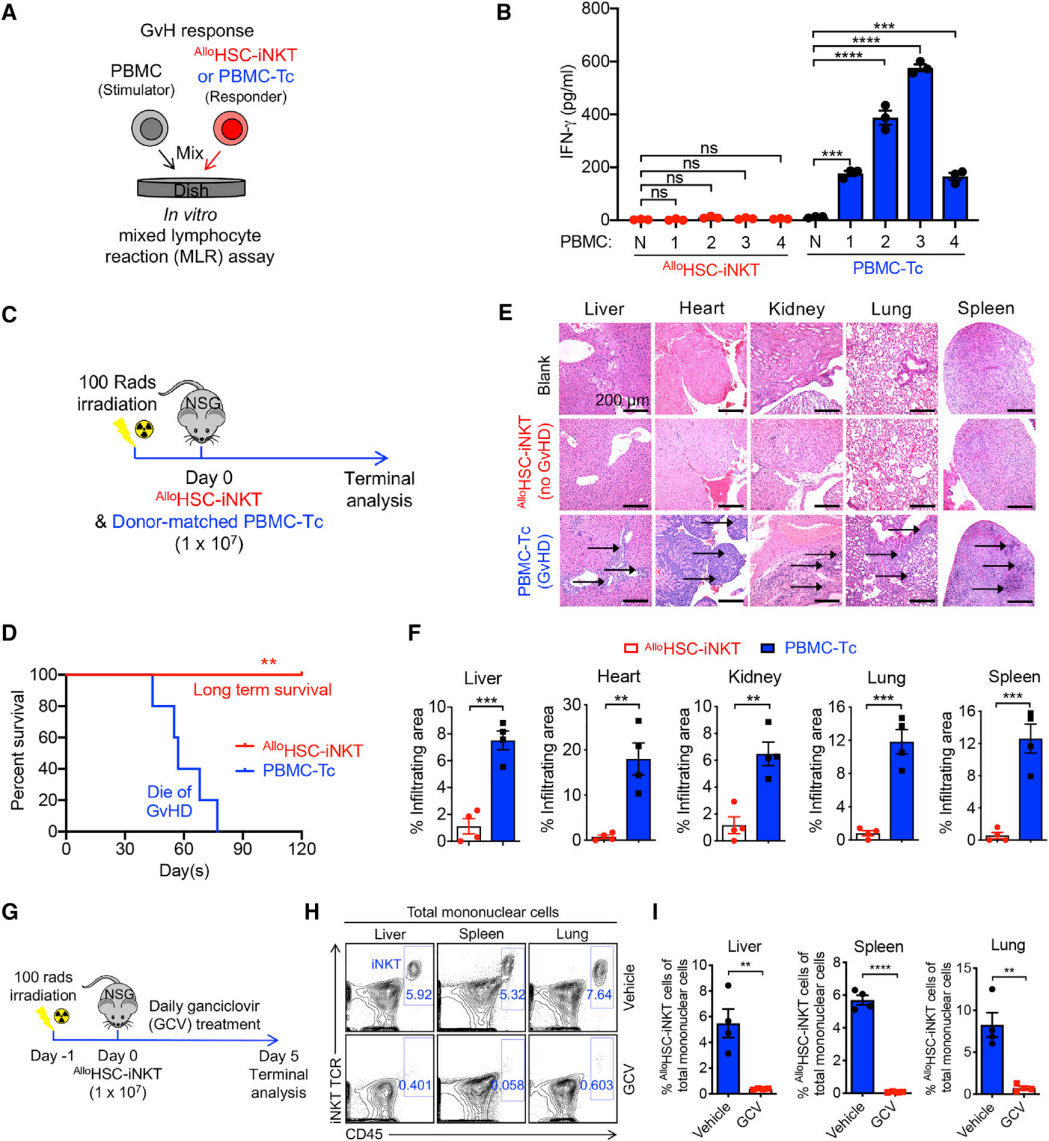
Safety study of ^Allo^HSC-iNKT cells (A and B) Studying the graft-versus-host (GvH) response of ^Allo^HSC-iNKT cells using an *in vitro* mixed lymphocyte reaction (MLR) assay. PBMC-Tc cells were included as a responder cell control. (A) Experimental design. PBMCs from four different healthy donors were used as stimulator cells. (B) ELISA analyses of IFN-γ production at day 4 (n = 4). N, no stimulator cells. (C–F) Studying the GvH response of ^Allo^HSC-iNKT cells using an NSG mouse xenograft model. Donor-matched PBMC-Tc cells were included as a control. (C) Experimental design. (D) Kaplan-Meier survival curves of experimental mice over time (n = 5). (E) H&E-stained tissue sections. Blank indicates tissue sections collected from control NSG mice receiving no adoptive cell transfer. Arrows point to mononuclear cell infiltrates. Scale bar, 200 μm. (F) Quantification of (E) (n = 4). (G–I) *In vivo* controlled depletion of ^Allo^HSC-iNKT cells via GCV treatment. GCV, ganciclovir. (G) Experimental design. (H) FACS detection of ^Allo^HSC-iNKT cells in the liver, spleen, and lung of NSG mice at day 5. (I) Quantification of (G) (n = 4). Representative of two experiments. Data are presented as the mean ± SEM. ns, not significant; ∗p < 0.05; ∗∗p < 0.01; ∗∗∗p < 0.001; ∗∗∗∗p < 0.0001 by one-way ANOVA (B), Student’s t test (F and I), or log rank (Mantel-Cox) test adjusted for multiple comparisons (D). See also Figure S3.

Graft-versus-host (GvH) response is the primary safety concern of an off-the-shelf allogeneic cell therapy.^4^ Since iNKT cells do not react to mismatched HLA molecules and protein alloantigens, these cells are not expected to mount GvH responses.^17^^,^^19^ To verify this safety feature of ^Allo^HSC-iNKT cells, we performed both *in vitro* and *in vivo* studies. In an *in vitro* mixed lymphocyte reaction (MLR) assay, in sharp contrast to conventional PBMC-Tc cells, ^Allo^HSC-iNKT cells did not react to all the mismatched healthy donor PBMCs tested, as evidence by their lack of IFN-γ production (Figures 5A and 5B). In an *in vivo* NSG mouse xenograft model, unlike PBMC-Tc cells that induced GvHD and killed experimental mice ∼2 months after PBMC-Tc cell transfer, ^Allo^HSC-iNKT cells did not cause GvHD and sustained long-term survival of experimental mice (Figures 5C and 5D). The lack of GvHD in ^Allo^HSC-iNKT cell engrafted experimental mice was confirmed by histology analysis showing healthy tissue structures without lymphocyte infiltrations; on the contrary, analyses of PBMC-Tc cell engrafted experimental mice showed severe tissue damages associated with heavy lymphocyte infiltrations (Figures 5E and 5F). To study the influence of CAR engineering, we analyzed the GvH response of ^Allo^BCAR-iNKT cells; BCAR-T cells were included as a control. Distinct from BCAR-T cells, ^Allo^BCAR-iNKT cells showed no response in the *in vitro* MLR assay (Figures S3A and S3B) and induced no GvHD in the human MM xenograft NSG mouse model (Figures S3C and S3D), indicating that CAR engineering and tumor encountering do not change the GvH-free safety feature of ^Allo^HSC-iNKT cells.

Besides GvHD risk, allogeneic cell therapy may confer other safety risks, including those common to cell-based cancer immunotherapy, such as cytokine release syndrome (CRS) and neurotoxicity.^63^ Although we did not observe that ^Allo^HSC-iNKT cells induced tissue toxicity in our NSG xenograft mouse models (Figures 5E and 5F), these safety studies may be limited by the utilized preclinical animal models.^63^ Additional safety controls may be necessary, especially for initial clinical development. We therefore have engineered a “safety switch” in ^Allo^HSC-iNKT cell products by incorporating a suicide gene (i.e., sr39TK) in the human iNKT TCR gene delivery vector, resulting in ^Allo^HSC-iNKT cells that are 100% labeled with the suicide gene (Figure 1A). In cell culture, addition of guanosine analog (ganciclovir [GCV]) effectively killed ^Allo^HSC-iNKT cells (Figure S3E); in an NSG mouse xenograft model, administration of GCV effectively depleted ^Allo^HSC-iNKT cells from all tissues examined (e.g., liver, spleen, and lung; Figures 5G–5I). Of note, GCV has been used clinically as a prodrug to induce sr39TK-mediated suicide effect in cellular products.^64^ Other alternative suicide switch systems (e.g., inducible Cas9 and truncated EGFR) can certainly be utilized.^4^^,^^65^, ^66^, ^67^

Taken together, our results show that ^Allo^HSC-iNKT cells are free of GvHD risk and can be equipped with an additional safety switch, making them suitable for off-the-shelf allogeneic cell therapy.

### Immunogenicity study of ^Allo^HSC-iNKT cells

**Figure 6:**
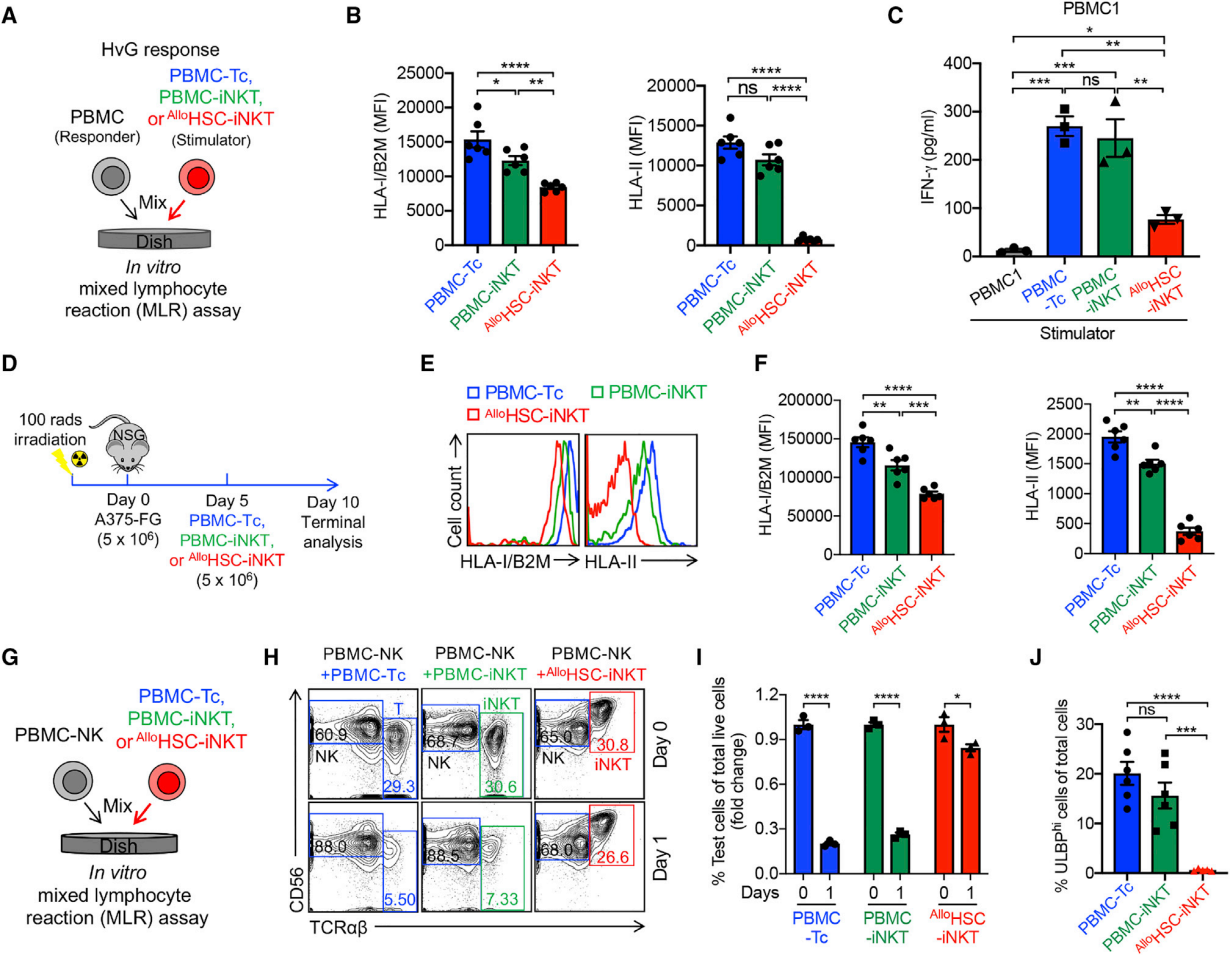
Immunogenicity study of ^Allo^HSC-iNKT cells (A–C) Studying allogenic T cell response against ^Allo^HSC-iNKT cells using an *in vitro* MLR assay. Irradiated ^Allo^HSC-iNKT cells (as stimulators) were co-cultured with donor-mismatched PBMC cells (as responders). Irradiated PBMC-iNKT and PBMC-Tc cells were included as stimulator cell controls. (A) Experimental design. PBMCs from three different healthy donors were used as responders. (B) FACS analyses of HLA-I and HLA-II expression on the indicated stimulator cells (n = 6). (C) ELISA analyses of IFN-γ production at day 4 (n = 3). (D–F) Studying HLA-I/II expression on ^Allo^HSC-iNKT cells *in vivo* in an A375-FG human melanoma xenograft NSG mouse model. PBMC-iNKT and PBMC-Tc cells were included as effector cell controls. (D) Experimental design. (E) FACS analyses of HLA-I/II expression on the indicated effector cells isolated from A375-FG solid tumors. (F) Quantification of (E) (n = 5). (G–J) Studying allogenic NK cell response against ^Allo^HSC-iNKT cells using an *in vitro* MLR assay. ^Allo^HSC-iNKT cells were co-cultured with donor-mismatched PBMC-NK cells. PBMC-iNKT and PBMC-Tc cells were included as controls. (G) Experimental design. (H) FACS analyses of the indicated cells at days 0 and 1. (I) Quantification of (H) (n = 3). (J) FACS analyses of ULBP expression on the indicated cells (n = 5–6). Representative of two (D–F) and three (A–C and G–J) experiments. Data are presented as mean ± SEM. ns, not significant; ∗p < 0.05; ∗∗p < 0.01; ∗∗∗p < 0.001; ∗∗∗∗p < 0.0001 by Student’s t test (I) or one-way ANOVA (B, C, F, and G). See also Figures S4–S6.

For allogeneic cell therapies, immunogenicity can be a concern, because allorejection by host T and NK cells can greatly limit the efficacy of therapeutic allogeneic cells.^68^ Host conventional CD8 and CD4 αβ T cells reject allogeneic cells by recognizing mismatched HLA-I and HLA-II molecules, respectively.^69^^,^^70^ In a classical *in vitro* MLR assay studying T-cell-mediated host-versus-graft (HvG) response via IFN-γ secretion reading, compared to endogenous conventional T and iNKT (i.e., PBMC-Tc and PBMC-iNKT) cells, ^Allo^HSC-iNKT cells triggered a significantly reduced HvG response (Figures 6A, 6C, and S4A). Our previous RNA-seq study made the interesting observation that compared to endogenous immune cells (i.e., conventional αβT, iNKT, γδT, and NK cells), ^Allo^HSC-iNKT cells globally downregulated the expression of many genes controlling the cell surface display of HLA-I and HLA-II molecules (Figure 2I). Flow cytometry analysis confirmed that compared to PBMC-Tc and PBMC-iNKT cells, ^Allo^HSC-iNKT cells expressed significantly reduced levels of HLA-I molecules and nearly undetectable levels of HLA-II molecules (Figures 6B and S4B), which may account for their resistance to T-cell-mediated HvG response (Figures 6C and S4A). Because IFNs can upregulate HLA-I expression,^71^ we studied HLA-I expression on ^Allo^HSC-iNKT cells under IFN-γ stimulation (Figure S5A). ^Allo^HSC-iNKT cells slightly upregulated surface HLA-I expression after IFN-γ stimulation; however, their overall surface HLA-I level still remained significantly lower than those of PBMC-Tc and PBMC-iNKT cells (Figures S5B and S5C). Because an *in vivo* inflammatory tumor microenvironment may upregulate the expression of HLA molecules on tumor-infiltrating immune cells (e.g., via IFN-γ),^72^ we also assessed HLA expression on ^Allo^HSC-iNKT cells in an A375-FG human melanoma xenograft NSG mouse model adapted from a previous study (Figures 3I). A375-FG melanoma cells were subcutaneously inoculated in NSG mice to form solid tumors, followed by injection of ^Allo^HSC-iNKT cells; PBMC-Tc and PBMC-iNKT cells were included as controls (Figure 6D). Flow cytometry analysis of tumor-infiltrating ^Allo^HSC-iNKT cells showed that these cells maintained low expression of HLA-I and HLA-II molecules at levels significantly lower than those of tumor-infiltrating PBMC-Tc and PBMC-iNKT cells (Figures 6E and 6F).

Next, we studied NK-cell-mediated allorejection. Host NK cells reject allogeneic cells through a double-trigger mechanism: (1) “missing self” (i.e., missing of matching HLA-I molecules on allogeneic cells) triggers the release of KIR inhibition, and (2) a “stress signal” (i.e., upregulated stress molecules on allogeneic cells) triggers the activation of NK activating receptors such as NKG2D.^52^^,^^73^^,^^74^ Because ^Allo^HSC-iNKT cells expressed low levels of HLA-I molecules, we wondered whether this might make ^Allo^HSC-iNKT cells susceptible to host NK rejection. Surprisingly, in an *in vitro* MLR assay studying NK-cell-mediated allorejection, compared to endogenous conventional T and iNKT (i.e., PBMC-Tc and PBMC-iNKT) cells, ^Allo^HSC-iNKT cells survived rejection by mismatched healthy donor PBMC-derived NK cells significantly better (Figures 6G–6I and S6); correspondingly, NK cells co-cultured with ^Allo^HSC-iNKT cells exhibited less upregulation of NK activation markers (i.e., CD107a) (Figures S4D and S4E). Flow cytometry analysis revealed that compared to PBMC-Tc and PBMC-iNKT cells, ^Allo^HSC-iNKT cells expressed much reduced and nearly undetectable levels of NKG2D ligands (i.e., ULBP; Figures 6J and S4C),^75^ which may be one of the possible mechanisms accounting for their resistance to NK-cell-mediated allorejection.

Collectively, these studies revealed a stable HLA-I/II^low^ phenotype of ^Allo^HSC-iNKT cells that may grant them advantage to resist host T-cell-mediated rejection compared to other healthy donor PBMC-derived allogeneic cell products; meanwhile, ^Allo^HSC-iNKT cells also expressed low levels of stress molecules such as NKG2D ligands, making them also resistant to host NK-cell-mediated allorejection. These “low immunogenicity” features of ^Allo^HSC-iNKT cells support their application for off-the-shelf cell therapy.

### Development of HLA-ablated universal HSC-iNKT (^U^HSC-iNKT) cells and derivatives

Although ^Allo^HSC-iNKT cells display a stable HLA-I/II^low^ phenotype (Figures 2I, 6B, 6E, 6F, and S4B), their residual HLA-I/II molecules may still make them susceptible to certain levels of host T-cell-mediated allorejection. We therefore explored further engineering of the ^Allo^HSC-iNKT cell products to achieve total ablation of their surface HLA-I/II molecules. Interestingly, this task can be accomplished by ablation of only two genes: (1) a *B2M* gene encoding the beta 2-microglobulin (B2M) that is required for the surface display of all types of HLA-I molecules,^69^ and (2) a *CIITA* gene encoding the class II transactivator (CIITA) that is required for the transcription of all types of HLA-II molecules.^70^ Ablation of *B2M* and *CIITA* genes can be achieved by using the powerful gene-editing tools like the CRISPR-Cas9/guide RNA (gRNA) system.^76^ We postulated that by combining iNKT TCR gene engineering and B2M/CIITA gene editing, we would produce HLA-ablated “universal” HSC-iNKT (^U^HSC-iNKT) cells totally resistant to host T-cell-mediated allorejection. We intended to perform both gene engineering and gene editing on the small numbers of starting HSCs upfront of the HSC-iNKT cell culture; this manufacturing design would save on the use of gene-engineering/editing materials (i.e., lentivector and CRISPR-Cas9/gRNA) that can be cost-limiting, and also enable the maximal gene engineering/editing efficiency that can be carried on into the final ^U^HSC-iNKT cell products. Similar to the generation of ^Allo^CAR-iNKT cell products (Figure 4A), a CAR engineering step can be incorporated after ^U^HSC-iNKT cell differentiation, resulting in HLA-ablated universal CAR-engineered HSC-iNKT (^U^CAR-iNKT) cell products.

**Figure 7:**
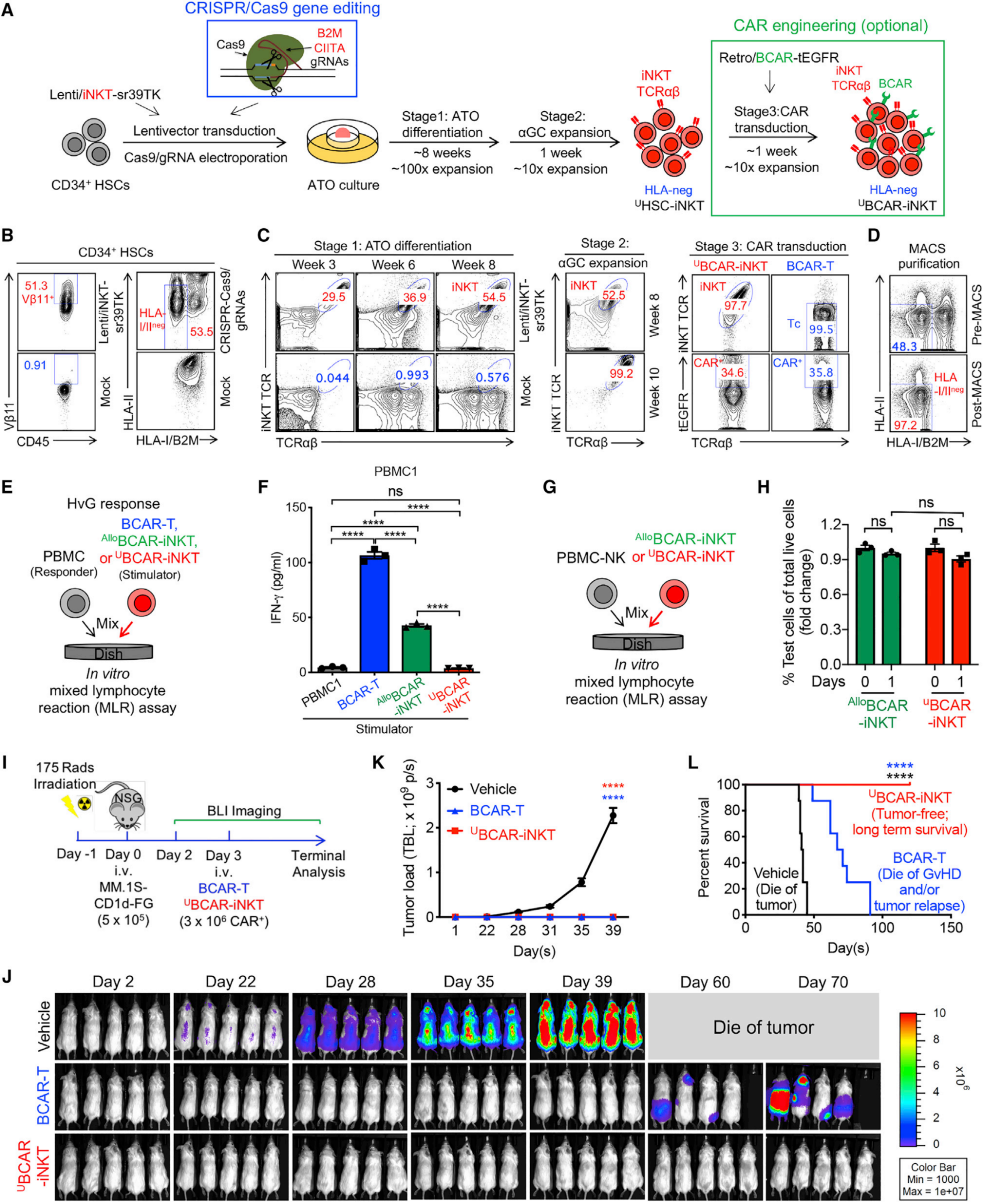
Development of HLA-ablated universal HSC-iNKT (^U^HSC-iNKT) cells and derivatives (A) Experimental design to generate ^U^HSC-iNKT and BCMA CAR-engineered ^U^HSC-iNKT (^U^BCAR-iNKT) cells. CRISPR, clusters of regularly interspaced short palindromic repeats; Cas 9, CRISPR-associated protein 9; gRNA, guide RNA; B2M, beta-2-microglobulin; CIITA, class II major histocompatibility complex transactivator. (B–D) FACS monitoring of ^U^HSC-iNKT and ^U^BCAR-iNKT cell generation. (B) Intracellular expression of iNKT TCR (identified as Vβ11^+^) and surface ablation of HLA-I/II (identified as HLA-I/B2M^−^HLA-II^−^) in CD34^+^ HSCs cells at day 5 (72 h after lentivector transduction and 48 h after CRISPR-Cas9 gene editing). (C) Generation of iNKT cells (identified as iNKT TCR^+^TCRαβ^+^ cells) during stage 1 ATO differentiation culture, stage 2 αGC expansion, and stage 3 CAR transduction. Healthy donor PBMC-T cells transduced with the same Retro/BCAR-tEGFR vector was included as a staining control (denoted as BCAR-T cells). (D) Purification of HLA-I/II-negative ^U^HSC-iNKT cells using MACS. (E and F) Studying allogenic T cell response against ^U^BCAR-iNKT cells using an *in vitro* MLR assay. Irradiated ^U^BCAR-iNKT cells (as stimulators) were co-cultured with donor-mismatched PBMCs (as responders). Irradiated ^Allo^BCAR-iNKT and conventional BCAR-T cells were included as stimulator cell controls. (E) Experimental design. PBMCs from three different healthy donors were used as responders. (F) ELISA analyses of IFN-γ production at day 4 (n = 3). (G and H) Studying allogenic NK cell response against ^U^HSC-iNKT cells using an *in vitro* MLR assay. ^U^HSC-iNKT cells were co-cultured with donor-mismatched PBMC-NK cells. ^Allo^HSC-iNKT cells were included as a control. (G) Experimental design. (H) FACS quantification of the indicated cells (n = 3). (I–L) Studying the *in vivo* antitumor efficacy of ^U^BCAR-iNKT cells in an MM.1S-CD1d-FG human multiple myeloma xenograft NSG mouse model. (I) Experimental design. (J) BLI images showing tumor loads in experimental mice over time. (K) Quantification of (J) (n = 5). (L) Kaplan-Meier survival curves of experimental mice over a period of 4 months after tumor challenge (n = 8). Mice were combined from two independent experiments. Representative of two (I–L) and three (B–H) experiments. Data are presented as mean ± SEM. ns, not significant; ∗∗∗∗p < 0.0001 by Student’s t test (H), one-way ANOVA (F and K), or log rank (Mantel-Cox) test adjusted for multiple comparisons (L). See also Figure S7.

CB or PBSC-derived CD34^+^ HSCs were transduced with the Lenti/iNKT-sr39TK lentivector; 24 h later, these HSCs were electroporated with a CRISPR-Cas9/B2M-CIITA-gRNAs complex (Figure 7A). The gene-engineering/editing efficiency was high; we routinely achieved over 50% lentivector transduction rate and over 50% HLA-I/II double-ablation rate (Figures 7B and S7B). The engineered HSCs were then put into the HSC-iNKT differentiation ATO culture for 8 weeks followed by 1 week of αGC expansion (Figures 7A); CRISPR-Cas9 gene editing did not interfere with HSC differentiation into iNKT cells and we obtained high yield of differentiated ^U^HSC-iNKT cells similar to that of ^Allo^HSC-iNKT cells (Figures 1C and 7C). CRISPR-Cas9 gene editing also did not interfere with the follow-up CAR engineering; we obtained an efficient BCMA-targeting CAR (BCAR) engineering rate similar to that of engineering PBMC-derived conventional BCAR-T cells (Figure 7C). The high HLA-I/II double-ablation rate of the starting HSCs was inherited by the resulting ^U^BCAR-iNKT cells (i.e., ∼50% of the resulting cells were HLA-I/II double negative); if needed, the HLA-I/II double-negative cells could be further enriched (to over 97%) using magnetic-activated cell sorting (MACS) via B2M and HLA-II magnetic beads labeling (Figures 7D).

Despite HLA-I/II ablation, ^U^BCAR-iNKT cells displayed a typical iNKT phenotype and a highly cytotoxic functionality similar to those of ^Allo^BCAR-iNKT cells (Figure S7A). As expected, ^U^BCAR-iNKT cells induced nearly undetectable T-cell-mediated alloresponse when mixed with mismatched healthy donor PBMCs (Figures 7E, 7F, and S7F); meanwhile, ^U^BCAR-iNKT cells maintained the resistance to NK-cell-mediated allorejection (Figures 7G, 7H, and S7G). Safety features of ^U^BCAR-iNKT cells resembled those of ^Allo^BCAR-iNKT cells: ^U^BCAR-iNKT cells did not mount GvH response (Figures S7C and S7D), and they were sensitive to sr39TK/GCV-induced suicide control (Figure S7E).

To study whether HLA ablation may impact the antitumor efficacy of ^U^BCAR-iNKT cells, we performed *in vitro* and *in vivo* antitumor assays. In an established *in vitro* MM tumor cell killing assay (Figure 4C), ^U^BCAR-iNKT cells effectively killed tumor cells at an efficacy comparable to that of ^Allo^BCAR-iNKT cells (Figures 4E, S7H, and S7I). Similar to ^Allo^BCAR-iNKT cells, ^U^BCAR-iNKT cells could also utilize an NK/TCR/CAR triple mechanisms targeting tumor cells (Figures 4E, 4F, S7I, and S7J), which may grant them an advantage over conventional BCAR-T cells to gain additional antitumor efficacy (Figure S7I), as well as to counteract the BCMA antigen escape that has been reported in conventional BCAR-T cell therapy clinical trials.^25^^,^^61^^,^^62^ In an established *in vivo* human MM xenograft NSG mouse model (Figure 4G), ^U^BCAR-iNKT-cell-treated animals achieved total tumor clearance and long-term survival, while BCAR-T-cell-treated animals only achieved partial tumor suppression that was followed by tumor relapse and GvHD development, leading to limited survival benefit (Figures 7I–7L).

Collectively, these studies support the generation of HLA-ablated universal HSC-iNKT cell products and derivatives (e.g., CAR-iNKT cell products) that are fully resistant to host T-cell-mediated allorejection and thereby may have improved *in vivo* persistence and antitumor efficacy.

## Discussion

Here, we report the generation and characterization of allogeneic HSC-engineered iNKT cells and derivatives. Using an *in vitro* HSC-iNKT differentiation cell culture, we generated ^Allo^HSC-iNKT cells that were of high yield and purity and had high antitumor efficacy, high safety profile (GvH-free and suicide control), and low immunogenicity (largely resistant to T- and NK-cell-mediated allorejection). These ^Allo^HSC-iNKT cells could be further engineered to express CAR, thereby enhancing their tumor-targeting capacity; these cells can also be further engineered to ablate their surface HLA molecules, thereby enhancing their resistance to host T-cell-mediated allorejection. Collectively, our studies have generated ^Allo^HSC-iNKT cells and demonstrated them as promising cell carriers for developing off-the-shelf cancer immunotherapy.

Development of allogeneic off-the-shelf cell therapies, many equipped with CARs, is becoming a fast-evolving frontier of cancer immunotherapy. Two major categories of such allogeneic cell products are based on engineering healthy-donor-derived conventional αβ T cells or NK cells.^14^^,^^68^^,^^69^^,^^77^ Because conventional αβ T cells risk inducing GvHD in allogeneic hosts due to HLA incompatibility, these T cells need to be gene edited to ablate endogenous TCR expression, usually by disrupting the *TRAC* or/and *TRBC* gene loci, to make them suitable for allogeneic cell therapy but meanwhile may also potentially increase manufacture complexity.^10^, ^11^, ^12^, ^13^ On the other hand, NK-based allogeneic cell products are considered of low GvHD risk and therefore do not require additional gene editing, but their *in vivo* clonal expansion and antitumor performance may be limited compared to that of conventional αβ T cells.^14^ Two such allogeneic cell products, conventional αβ T-cell-based universal CD19-CAR-engineered T cells (UCART19) and NK-cell-based CB-derived CD19-CAR-engineered NK cells, were recently tested in phase 1 clinical trials treating CD19^+^ B cell malignancies.^10^^,^^78^ These studies reported the feasibility, certain antileukemic activity, and manageable safety profile of the two cell products, showing an encouraging step forward for the field of allogeneic cell therapy.^10^^,^^78^

Engineering unconventional innate-type T cells (e.g., iNKT, γδ T, and mucosal-associated invariant T cells) that have potent antitumor capacity while being free of GvHD risk represents another promising direction for developing allogeneic cell therapy for cancer, especially for solid tumors.^25^^,^^79^, ^80^, ^81^, ^82^, ^83^ In particular, iNKT-cell-based cancer immunotherapy has attracted considerable attention. A preclinical study reported the enhanced anti-lymphoma activity of CAR19-engineered iNKT cells compared to conventional CAR19-T cells.^25^ Another preclinical study reported the potent antitumor efficacy to neuroblastoma and no GvHD risk of CAR.GD2-engineered iNKT cells compared to conventional CAR.GD2-T cells.^80^ A recent clinical trial testing autologous GD2.CAR-engineered iNKT cells also showed safety and certain efficacy in patients with relapsed or refractory neuroblastoma.^79^ These studies suggest the therapeutical potential of iNKT-based cell products and support the development of such cell products for allogeneic cell therapy for cancer even solid tumors.

The *in vitro* HSC-iNKT differentiation cell culture was robust and of high yield (Figure 1G). The resulting ^Allo^HSC-iNKT cell products were of high purity and nearly free of bystander conventional αβ T cells (Figure 1D), which may allow these cell products to be directly used for allogeneic cell therapy without an additional purification step; if necessary, commercially available human iNKT cell isolation reagents (e.g., human anti-iNKT microbeads from Miltenyi) can be used for further purification. Notably, we detected no expression of endogenous TCRs in ^Allo^HSC-iNKT cells (Figure 1F), suggesting an induction of allelic exclusion by transgenic iNKT TCRs as previously reported.^27^^,^^28^ The robustness, high yield, and high purity of ^Allo^HSC-iNKT cell products will facilitate their next-stage translational and clinical development.

The antitumor efficacy of ^Allo^HSC-iNKT cells is promising. These cells display a typical iNKT phenotype and functionality; they co-express memory T cell and NK cell markers, express high levels of inflammatory tissue/tumor homing markers, and produce high levels of cytokines and cytotoxic molecules, outperforming T and NK cells (Figures 2 and 3). Interestingly, compared to endogenous cells (i.e., T, NK, and iNKT cells), ^Allo^HSC-iNKT cells express exceedingly high levels of NK activating receptors and low levels of NK inhibitory receptors (Figure 2G), which is associated with their superior antitumor NK function *in vitro* and *in vivo* (Figure 3). In addition, the NK/TCR/CAR tumor-targeting triple mechanisms of ^Allo^HSC-iNKT cells and their derivatives grant these cells a stronger antitumor efficacy (Figures 4D–4F, 7I–7J, and S6G–S6I) and may enable them to counteract tumor antigen escape, which has been observed in T-cell-based cancer therapies.^4^^,^^25^^,^^61^^,^^62^ Overall, in both the human blood cancer (i.e., MM) and human solid tumor (i.e., melanoma) preclinical mouse xenograft models utilized in this study, ^Allo^HSC-iNKT cells or their CAR derivatives (i.e., ^Allo^BCAR-iNKT and ^U^BCAR-iNKT cells) showed an enhanced antitumor efficacy compared to healthy donor PBMC-derived NK or CAR-engineered conventional αβ T cells, highlighting their cancer therapy potential (Figures 3I–3K, S1H, S1I, 4G–4N, S2E, and 7I–7L). Notably, a synthetic iNKT cell antagonist, αGC, has been demonstrated to specifically stimulate and expand iNKT cells in both preclinical and clinical studies; αGC is clinically available and may be used as a “designer stimulator” to enhance the *in vivo* performance of ^Allo^HSC-iNKT cells.^18^^,^^23^^,^^84^, ^85^, ^86^

The safety of ^Allo^HSC-iNKT cells is appealing. In our studies, ^Allo^HSC-iNKT cells showed no GvH responses against multiple random healthy donor PBMCs in an *in vitro* MLR assay (Figures 5A, 5B, S3A, and S3B) and no GvHD *in vivo* in multiple human tumor xenograft NSG mouse models (Figures 5C–5F, S3C, and S3D), consistent with their iNKT cell nature and high purity (Figure 1).^16^ A suicide switch (e.g., sr39TK/GCV) can also be incorporated into ^Allo^HSC-iNKT cells to provide an additional safety control (Figure 5G, 5I, and S3E). The high safety of ^Allo^HSC-iNKT cells strongly support their allogeneic application.

A serendipitous feature of ^Allo^HSC-iNKT cells is their significantly lower immunogenicity compared to PBMC-derived endogenous immune cells (i.e., αβ T, iNKT, γδ T, and NK cells). ^Allo^HSC-iNKT cells express reduced levels of HLA-I molecules and nearly undetectable levels of HLA-II molecules, which seems to be genomically programmed (Figure 2I) and stable through the *in vitro* culture and *in vivo* persistence even within the tumor microenvironment (Figures 6A–6F); this feature may allow these cells to resist allorejection by host T cells and thereby alleviate the need for additional HLA gene editing or intense host T cell depletion preconditioning treatment (e.g., CD52 antibody treatment).^10^^,^^13^ Meanwhile, ^Allo^HSC-iNKT cells also express reduced levels of NK activating receptor ligands (e.g., ULBP) (Figure 6J); this feature may allow these cells to also resist allorejection by host NK cells and increase their suitability for allogeneic cell therapy (Figures 6G–6I). The biological regulations resulting in this low-immunogenicity feature of ^Allo^HSC-iNKT cells remain to be illustrated; nonetheless, such biological regulations do not seem to interfere with the production or antitumor efficacy of ^Allo^HSC-iNKT cells both *in vitro* and *in vivo* (Figures 3, 4, and 7).

The ^Allo^HSC-iNKT cell production platform is robust and versatile, allowing the plug-in of additional engineering approaches. In our studies, we demonstrated the successful generation of CAR-engineered or/and HLA-I/II-ablated ^Allo^HSC-iNKT cells by incorporating additional CAR gene engineering and CRISPR-Cas9/B2M-CIITA-gRNAs gene-editing steps (Figures 4 and 7). These additional engineering approaches did not interfere with the production and antitumor function of ^Allo^HSC-iNKT cells, opening up the possibility of developing more advanced ^Allo^HSC-iNKT cell products. For example, incorporation of multiple tumor-targeting molecules (e.g., CARs and TCRs) and functional enhancement factors (e.g., overexpression of immune enhancement genes like IL-15, and ablation of immune inhibitory genes like PD-1) may improve the cancer therapy potential of ^Allo^HSC-iNKT cell products.^87^^,^^88^

### Limitations of the study

Despite their promise, current ^Allo^HSC-iNKT cell products confront certain limitations that may be further improved; the manufacturing process can benefit from switching to a feeder-free culture system that will greatly simplify and accelerate the clinical and commercial development, the sr39TK/GCV suicide switch can be replaced with an alternative suicide switch system (e.g., inducible Cas9 or truncated EGFR) that is less immunogenic and cell-cycle dependent,^4^^,^^65^, ^66^, ^67^^,^^89^ an *HLA-E* transgene can also be incorporated into the ^Allo^HSC-iNKT cell products to further increase their resistance to host NK-cell-mediated allorejection,^73^^,^^90^ and pluripotent stem cells (i.e., embryonic stem cells and induced pluripotent stem cells [iPSCs]) may be utilized as an alternative “unlimited” cell source to derive HSCs for the generation of ^Allo^HSC-iNKT cells.^24^ Further exploration of ^Allo^HSC-iNKT cells as allogeneic cell carriers for developing off-the-shelf cell therapy for the treatment of cancer, especially solid tumors, will certainly be an interesting direction for future study.

## STAR★Methods

### Key resources table

**Table:** 

REAGENT or RESOURCE	SOURCE	IDENTIFIER
**Antibodies**		

Anti-human IFN-γ (ELISA, capture)	BD Biosciences	CAT#551221, RRID: AB_394099
Anti-human IFN-γ (ELISA, detection)	BD Biosciences	CAT#554550, RRID: AB_395472
Anti-human TNFα (ELISA, capture)	BD Biosciences	CAT#551220, RRID: AB_394098
Anti-human TNFα (ELISA, detection)	BD Biosciences	CAT#554511, RRID: AB_395442
Anti-human IL-2 (ELISA, detection)	BD Biosciences	CAT#554563; RRID: AB_398570
Anti-human IL-2 (ELISA, detection)	BD Biosciences	CAT#555040; RRID: AB_395666
Anti-human IL-4 (ELISA, capture)	BD Biosciences	CAT#554515; RRID: AB_398567
Anti-human IL-4 (ELISA, detection)	BD Biosciences	CAT#554483; RRID: AB_395422
Anti-human CD45 (Clone H130)	Biolegend	CAT#304026, RFID: AB_893337
Anti-human TCRαβ (Clone I26)	Biolegend	CAT#306716, RRID: AB_1953257
Anti-human CD4 (Clone OKT4)	Biolegend	CAT#317414, RRID: AB_571959
Anti-human CD8 (Clone SK1)	Biolegend	CAT#344714, RRID: AB_2044006
Anti-human CD45RO (Clone UCHL1)	Biolegend	CAT#304216, RRID: AB_493659
Anti-human CD161 (Clone HP-3G10)	Biolegend	CAT#339928, RRID: AB_2563967
Anti-human CD69 (Clone FN50)	Biolegend	CAT#310914, RRID: AB_314849
Anti-human CD56 (Clone HCD56)	Biolegend	CAT#318304, RRID: AB_604100
Anti-human CD62L (Clone DREG-56)	Biolegend	CAT#304822, RRID: AB_830801
Anti-human CD14 (Clone HCD14)	Biolegend	CAT#325608, RRID: AB_830681
Anti-human CD11b (Clone ICRF44)	Biolegend	CAT#301330, RRID: AB_2561703
Anti-human CD11c (Clone N418)	Biolegend	CAT#337234, RRID: AB_2566656
Anti-human CD1d (Clone 51.1)	Biolegend	CAT#350308, RRID: AB_10642829
Anti-human CCR4 (Clone L291H4)	Biolegend	CAT#359409, RRID: AB_2562430
Anti-human CCR5 (Clone HEK/1/85a)	Biolegend	CAT#313705, RRID: AB_345305
Anti-human CXCR3 (Clone G025H7)	Biolegend	CAT#306513, RRID: AB_2089652
Anti-human NKG2D (Clone 1D11)	Biolegend	CAT#320812, RRID: AB_2234394
Anti-human DNAM-1 (Clone 11A8)	Biolegend	CAT#338312, RRID: AB_2561952
Anti-human CD158 (KIR2DL1/S1/S3/S5) (Clone HP-MA4)	Biolegend	CAT#339510, RRID: AB_2565577
Anti-human IFN-γ (Clone B27)	Biolegend	CAT#506518, RRID: AB_2123321
Anti-human granzyme B (Clone QA16A02)	Biolegend	CAT#372204, RRID: AB_2687028
Anti-human perforin (Clone dG9)	Biolegend	CAT#308126, RRID: AB_2572049
Anti-human TNFα (Clone Mab11)	Biolegend	CAT#502912, RRID: AB_315264
Anti-human IL-2 (Clone MQ1-17H12)	Biolegend	CAT#500341, RRID: AB_2562854
Anti-human HLAE (Clone 3D12)	Biolegend	CAT#342606, RRID: AB_342606
Anti-human β2-microglobulin (B2M) (Clone 2M2)	Biolegend	CAT#316312, RRID: AB_10641281
Anti-human HLA-DR (Clone L243)	Biolegend	CAT#307618, RRID: AB_493586
Anti-human TCR Vδ2 (Clone B6)	Biolegend	CAT#331417, RRID: AB_2687323
Anti-human CD107a (Clone H4A3)	Biolegend	CAT#328641, RRID: AB_2565965
Anti-human CD34 (Clone 581)	BD Biosciences	CAT#555822, RRID: AB_396151
Anti-human TCR Vα24-Jβ18 (Clone 6B11)	BD Biosciences	CAT#552825, RRID: AB_394478
Anti-human ULBP-2,5,6 (Clone 165903)	R&D Systems	CAT#FAB1298A, RRID: AB_ 2257142
Anti-human Vβ11	Beckman-Coulter	CAT#A66905
Human Fc Receptor Blocking Solution (TrueStain FcX)	Biolegend	CAT#422302
Mouse Fc Block (anti-mouse CD16/32)	BD Biosciences	CAT#553142, RRID: AB_394657
β-2-Microglobulin Antibody (Clone BBM.1)	Santa Cruz Biotechnology	CAT#sc-13565
LEAF purified anti-human CD1d antibody (Clone 51.1)	Biolegend	CAT#350304
LEAF purified Mouse IgG2b, k isotype ctrl (Clone MG2b-57)	Biolegend	CAT#401212
LEAF purified anti-human NKG2D antibody (Clone 1D11)	Biolegend	CAT#320810, RRID: AB_2133276
LEAF purified anti-human DNAM-1 antibody (Clone DX11)	BD Biosciences	CAT#559786, RRID: AB_397327
Mouse IgG1, κ isotype control antibody (Clone MOPC-21)	Biolegend	CAT#400124

**Bacterial and virus strains**		

Lenti/iNKT-sr39TK	This paper	N/A
Lenti/BCAR-iNKT-sr39TK	This paper	N/A
Lenti/FG	This paper	N/A
Lenti/CD1d	This paper	N/A
Retro/BCMA-CAR-tEGFR	This paper	N/A

**Biological samples**		

Human peripheral blood mononuclear cells (PBMCs)	UCLA	N/A
Human cord blood CD34+ hematopoietic stem and progenitor cells (HSCs)	UCLA	N/A
Human multiple myeloma patient bone marrow samples	UCLA	N/A
G-CSF-mobilized peripheral blood units	CCHMC	CAT#M001F-GCSF-3
G-CSF-mobilized leukopak	HemaCare	CAT#M001CLPG-4-KIT
Cord Blood Cryo CD34	HemaCare	CAT#CB34C-3

**Chemicals, peptides, and recombinant proteins**		

Streptavidin-HRP conjugate	Invitrogen	CAT#SA10001
IFN-γ (ELISA, standard)	eBioscience	CAT#29-8319-65
TNFα (ELISA, standard)	eBioscience	CAT#29-8329-65
IL-2 (ELISA, standard)	eBioscience	CAT#29-8029-65
IL-4 (ELISA, standard)	eBioscience	CAT#39-8049-65
IL-17 (ELISA, standard)	eBioscience	CAT#29-8179-65
Tetramethylbenzidine (TMB)	KPL	CAT#5120-0053
Ganciclovir (GCV)	Sigma	CAT#ADV465749843
α-Galactosylceramide (KRN7000)	Avanti Polar Lipids	SKU#867000P-1mg
Zoledronate	Sigma-Aldrich	CAT#SML0223
Recombinant human IL-2	Peprotech	CAT#200-02
Recombinant human IL-3	Peprotech	CAT#200-03
Recombinant human IL-7	Peprotech	CAT#200-07
Recombinant human IL-15	Peprotech	CAT#200-15
Recombinant human Flt3-Ligand	Peprotech	CAT#300-19
Recombinant human SCF	Peprotech	CAT#300-07
Recombinant human TPO	Peprotech	CAT#300-18
Recombinant human GM-CSF	Peprotech	CAT#300-03
L-ascorbic acid 2-phosphate	Sigma	CAT#A8960-5G
B27™ Supplement (50X), serum free	ThermoFisher	CAT#17504044
Cas9-NLS purified protein	UC Berkeley	N/A
X-VIVO 15 Serum-free Hematopoietic Cell Medium	Lonza	CAT#04-418Q
RPMI1640 cell culture medium	Corning Cellgro	CAT#10-040-CV
DMEM cell culture medium	Corning Cellgro	CAT#10-013-CV
Fetal Bovine Serum (FBS)	Sigma	CAT#F2442
MACS BSA stock solution	Miltenyi	CAT#130-091-376
30% BSA	Gemini	CAT#50-753-3079
Penicillin-Streptomycine-Glutamine (P/S/G)	GIBCO	CAT#10378016
Penicillin: streptomycin (pen:strep) solution (P/S)	Gemini Bio-products	CAT#400-109
MEM non-essential amino acids (NEAA)	GIBCO	CAT#11140050
HEPES Buffer Solution	GIBCO	CAT#15630056
Sodium Pyruvate	GIBCO	CAT#11360070
Beta-Mercaptoethanol	Sigma	SKU#M6250
Normocin	Invivogen	CAT#ant-nr-2
Fixable Viability Dye eFluor506	affymetrix eBioscience	CAT#65-0866-14
Cell Fixation/Permeabilization Kit	BD Biosciences	CAT#554714
RetroNectin recombination human fibronectin fragment, 2.5mg	Takara	CAT#T100B
10% neutral-buffered formalin	Richard-Allan Scientific	CAT#5705
D-Luciferin	Caliper LIfe Science	CAT#XR-1001
Isoflurane	Zoetis	CAT#50019100
Phosphate Buffered Saline (PBS) pH 7.4 (1X)	GIBCO	CAT#10010-023
Formaldehyde	Sigma-Aldrich	CAT#F8775
Golgistop Protein Transport Inhibitor	BD Biosciences	CAT#554724
Phorbol-12-myristate-13-acetate (PMA)	Calbiochem	CAT#524400
Ionomycin, Calcium salt, Streptomyces conglobatus	Calbiochem	CAT#407952

**Critical commercial assays**		

Human NK Cell Isolation Kit	Miltenyi Biotec	CAT#130-092-657
Human CD34 MicroBeads Kit	Miltenyi Biotec	CAT#130-046-703
Human CD14 MicroBeads Kit	Miltenyi Biotec	CAT#130-050-201
Human Anti-iNKT MicroBeads	Miltenyi Biotec	CAT#130-094-842
Human Anti-HLA-DR MicroBeads	Miltenyi Biotec	CAT#130-046-101
Fixation/Permeabilization Solution Kit	BD Sciences	CAT#55474
Amaxa™ P3 Primary Cell 4D-Nucleofector™ X Kit S	Lonza	CAT#V4XP-3032
Dynabeads Human T-Activator CD3/CD28	ThermoFisher	CAT#111.61D
miRNeasy Mini Kit	QIAGEN	CAT#217004
Chromium single cell V(D)J enrichment kit, human T cell	10 x Genomics	CAT#1000005
Cryostor cell cryopreservation media	Sigma	CAT#C2874-100ML
Human IL-17A ELISA MAX Deluxe Kit	Biolegend	CAT#433915

**Deposited data**		

Deep RNA sequencing	This paper	Gene Expression Omnibus Database: GSE164425
Single cell TCR sequencing	This paper	Gene Expression Omnibus Database: GSE164500

**Experimental models: Cell lines**		

Human multiple myeloma (MM) cell line MM.1S	ATCC	CRL-2974
Human chronic myelogenous leukemia cancer cell line K562	ATCC	CCL-243
Human melanoma cell line A375	ATCC	CRL-1619
Human adenocarcinoma cell line PC3	ATCC	CRL-1435
Human mucoepidermoid pulmonary carcinoma H292	ATCC	CRL-1848
*Mus musculus* Leukemia packaging cell PG13	ATCC	CRL-10686
Human multiple myeloma (MM) cell line MM.1S-FG	This paper	N/A
Human multiple myeloma (MM) cell line MM.1S-CD1d-FG	This paper	N/A
Human chronic myelogenous leukemia cancer cell line K562-FG	This paper	N/A
Human adenocarcinoma cell line PC3-FG	This paper	N/A
Human mucoepidermoid pulmonary carcinoma H292-FG	This paper	N/A
Human melanoma cell line A375-FG	This paper	N/A
*Mus musculus* Leukemia packaging cell PG13-BCAR-tEGFR	This paper	N/A
Mouse bone marrow derived stromal cell line MS5-hDLL4	Amelie et al., 2019	N/A

**Experimental models: Organisms/strains**		

NOD.Cg-Prkdcscid Il2rgtm1Wjl/SzJ (NSG)	The Jackson Laboratory	Stock #: 005557

**Oligonucleotides**		

gRNA (B2M): CGCGAGCACAGCUAA GGCCA	Synthego	N/A
gRNA (CIITA): GAUAUUGGCAUAAGC CUCCC	Synthego	N/A

**Recombinant DNA**		

Vector: parental lentivector pMNDW	N/A	N/A
Vector: parental retrovector pMP71	N/A	N/A

**Software and algorithms**		

FlowJo Software	FlowJo	https://www.flowjo.com/solutions/flowjo/downloads
Living Imaging 2.50 software	Xenogen/PerkinElmer	https://www.perkinelmer.com/lab-products-and-services/resources/in-vivo-imaging-software-downloads.html
AURA imaging software	Spectral Instruments Imaging	https://spectralinvivo.com/software/
I-control 1.7 Microplate Reader Software	Tecan	https://www.selectscience.net/tecan/i-control-microplate-reader-software/81307
ImageJ	ImageJ	https://imagej.net/Downloads
Prism 6	Graphpad	https://www.graphpad.com/scientific-software/prism/
MATLAB	The MathWorks, Inc	https://www.mathworks.com/products/matlab.html
R	R	http://www.R-project.org/

### Resource availability

#### Lead contact

Further information and requests for new reagents generated in this study may be directed to, and will be fulfilled by the Lead Contact, Lili Yang (liliyang@ucla.edu).

#### Materials availability

All unique/stable reagents generated in this study are available from the Lead Contact with a completed Materials Transfer Agreement.

### Experimental model and subject details

#### Mice

NOD.Cg-Prkdc^SCID^Il2rg^tm1Wjl^/SzJ (NOD/SCID/IL-2Rγ^−/−^, NSG) mice were maintained in the animal facilities of the University of California, Los Angeles (UCLA). 6-10 weeks old mice were used for all experiments unless otherwise indicated. All animal experiments were approved by the Institutional Animal Care and Use Committee of UCLA. All mice were bred and maintained under specific pathogen-free conditions, and all experiments were conducted in accordance with the animal care and use regulations of the Division of Laboratory Animal Medicine (DLAM) at the UCLA.

#### Cell Lines

The MS5-DLL4 murine bone marrow derived stromal cell line was obtained from Dr. Gay Crooks’ lab (UCLA). Human multiple myeloma cancer cell line MM.1S, chronic myelogenous leukemia cancer cell line K562, melanoma cell line A375, lung carcinoma cell line H292, and prostate cancer cell line PC3 were purchased from the American Type Culture Collection (ATCC). MM.1S cells were cultured in R10 medium. K562 cells were cultured in C10 medium. A375, H292, and PC3 were cultured in D10 medium.

To make stable tumor cell lines overexpressing human CD1d, and/or firefly luciferase and enhanced green fluorescence protein (Fluc-EGFP) dual-reporters, the parental tumor cell lines were transduced with lentiviral vectors encoding the intended gene(s)^28^. 72h post lentivector transduction, cells were subjected to flow cytometry sorting to isolate gene-engineered cells for making stable cell lines. Six stable tumor cell lines were generated for this study, including MM.1S-FG, MM.1S-CD1d-FG, A375-FG, PC3-FG, H292-FG, and K562-FG.

#### Human CD34^+^ Hematopoietic Stem Cells (HSCs), Periphery Blood Mononuclear Cells (PBMCs), and Patient Bone Marrow Samples

Cord blood cells were purchased from HemaCare. G-CSF-mobilized healthy donor peripheral blood cells were purchased from HemaCare or Cincinnati Children’s Hospital Medical Center (CCHMC). Human CD34^+^ HSCs were isolated through magnetic-activated cell sorting using ClinMACs CD34^+^ microbeads (Miltenyi Biotech). Cells were cryopreserved in Cryostor CS10 (Sigma) using CoolCell (BioCision), and were frozen in liquid nitrogen for all experiments and long-term storage. Healthy donor human PBMCs were obtained from the UCLA/CFAR Virology Core Laboratory, without identification information under federal and state regulations. Patient bone marrow samples were collected following UCLA IRB approval (IRB#15-000062).

#### Media and Reagents

α-Galactosylceramide (αGC, KRN7000) was purchased from Avanti Polar Lipids. Recombinant human IL-2, IL-3, IL-4, IL-7, IL-15, Flt3-Ligand, Stem Cell Factor (SCF), Thrombopoietin (TPO), and Granulocyte-Macrophage Colony-Stimulating Factor (GM-CSF) were purchased from Peprotech. Ganciclovir (GCV) was purchased from Sigma.

X-VIVO 15 Serum-free Hematopoietic Cell Medium was purchased from Lonza. RPMI 1640 and DMEM cell culture medium were purchased from Corning Cellgro. Fetal bovine serum (FBS) was purchased from Sigma. Medium supplements, including Penicillin-Streptomycine-Glutamine (P/S/G), MEM non-essential amino acids (NEAA), HEPES Buffer Solution, and Sodium Pyruvate, were purchased from GIBCO. Beta-Mercaptoethanol (β-ME) was purchased from Sigma. Normocin was purchased from InvivoGen. Complete lymphocyte culture medium (denoted as C10 medium) was made of RPMI 1640 supplemented with FBS (10% vol/vol), P/S/G (1% vol/vol), MEM NEAA (1% vol/vol), HEPES (10 mM), Sodium Pyruvate (1 mM), β-ME (50 mM), and Normocin (100 mg/ml). Medium for culturing human MM.1S tumor cell line (denoted as R10 medium) was made of RPMI 1640 supplemented with FBS (10% vol/vol) and P/S/G (1% vol/vol). Adherent cell culture medium (denoted as D10 medium) was made of DMEM supplemented with FBS (10% vol/vol) and P/S/G (1% vol/vol).

### Method details

#### Lentiviral and Retroviral Vectors

Lentiviral vectors used in this study were all constructed from a parental lentivector pMNDW as previously described^28^. The Lenti/iNKT-sr39TK vector was constructed by inserting into pMNDW vector a synthetic tricistronic gene encoding human iNKT TCRα-F2A-TCRβ-P2A-sr39TK; the Lenti/FG vector was constructed by inserting into pMNDW a synthetic bicistronic gene encoding Fluc-P2A-EGFP; the Lenti/CD1d vector was constructed by inserting into pMNDW a synthetic gene encoding human CD1d. The synthetic gene fragments were obtained from GenScript and IDT. Lentiviruses were produced using HEK293T cells, following a standard calcium precipitation protocol and an ultracentrifigation concentration protocol as previously described^27^^,^^28^. Lentivector titers were measured by transducing HT29 cells with serial dilutions and performing digital qPCR, following established protocols^27^^,^^28^.

The Retro/BCAR-tEGFR vector was constructed by inserting into the parental MP71 vector a synthetic gene encoding human BCMA scFV-41BB-CD3ζ-P2A-tEGFR. The synthetic gene fragments were obtained from IDT. Vsv-g-pseudotyped Retro/BCAR-tEGFR retroviruses were generated by transfecting HEK293T cells following a standard calcium precipitation protocol^27^^,^^28^; the viruses were then used to transduce PG13 cells to generate a stable retroviral packaging cell line producing Retro/BCAR-tEGFR retroviruses (denoted as PG13-BCAR-tEGFR cell line). For retrovirus production, the PG13-BCAR-tEGFR cells were seeded at a density of 8 × 10^5^ cells per ml in D10 medium and cultured in a 15 cm-dish (30 mL per dish) for 2 days; virus supernatants were then harvested and stored at −80°C for future use.

#### Antibodies and Flow Cytometry

All flow cytometry stains were performed in PBS for 15 min at 4°C. The samples were stained with Fixable Viability Dye eFluor506 (e506) mixed with Mouse Fc Block (anti-mouse CD16/32) or Human Fc Receptor Blocking Solution (TrueStain FcX) prior to antibody staining. Antibody staining was performed at a dilution according to the manufacturer’s instructions. Fluorochrome-conjugated antibodies specific for human CD45 (Clone H130), TCRαβ (Clone I26), CD4 (Clone OKT4), CD8 (Clone SK1), CD45RO (Clone UCHL1), CD161 (Clone HP-3G10), CD69 (Clone FN50), CD56 (Clone HCD56), CD62L (Clone DREG-56), CD14 (Clone HCD14), CD11b (Clone ICRF44), CD11c (Clone N418), CD1d (Clone 51.1), CCR4 (Clone L291H4), CCR5 (Clone HEK/1/85a), CXCR3 (Clone G025H7), NKG2D (Clone 1D11), DNAM-1 (Clone 11A8), CD158 (KIR2DL1/S1/S3/S5) (Clone HP-MA4), IFN-γ (Clone B27), granzyme B (Clone QA16A02), perforin (Clone dG9), TNF-α (Clone Mab11), IL-2 (Clone MQ1-17H12), HLAE (Clone 3D12), β2-microglobulin (B2M) (Clone 2M2), HLA-DR (Clone L243), TCR Vδ2 (Clone B6) were purchased from BioLegend; Fluorochrome-conjugated antibodies specific for human CD34 (Clone 581) and TCR Vɑ24-Jβ18 (Clone 6B11) were purchased from BD Biosciences; Fluorochrome-conjugated antibodies specific for human Vβ11 was purchased from Beckman-Coulter; Fluorochrome-conjugated antibodies specific for human ULBP-2,5,6 (Clone 165903) was purchased from R&D Systems. Human Fc Receptor Blocking Solution (TrueStain FcX) was purchased from Biolegend, and Mouse Fc Block (anti-mouse CD16/32) was purchased from BD Biosciences. Fixable Viability Dye e506 were purchased from Affymetrix eBioscience. Intracellular cytokines were stained using a Cell Fixation/Permeabilization Kit (BD Biosciences). Stained cells were analyzed using a MACSQuant Analyzer 10 flow cytometer (Miltenyi Biotech). FlowJo software was utilized to analyze the data.

#### Enzyme-Linked Immunosorbent Cytokine Assays (ELISA)

The ELISAs for detecting human cytokines were performed following a standard protocol from BD Biosciences. Supernatants from co-culture assays were collected and assayed to quantify IFN-γ, TNF-α, IL-2, IL-4, and IL-17. The capture and biotinylated pairs for detecting cytokines were purchased from BD Biosciences. The streptavidin-HRP conjugate was purchased from Invitrogen. Human cytokine standards were purchased from eBioscience. Tetramethylbenzidine (TMB) substrate was purchased from KPL. The samples were analyzed for absorbance at 450 nm using an Infinite M1000 microplate reader (Tecan).

#### *In Vitro* Generation of Allogeneic HSC-Engineered iNKT (^Allo^HSC-iNKT) Cells

Frozen-thawed human CD34^+^ HSCs were revived in HSC-culture medium composed of X-VIVO 15 Serum-free Hematopoietic Cell Medium supplemented with SCF (50 ng/ml), FLT3-L (50 ng/ml), TPO (50 ng/ml), and IL-3 (10 ng/ml) for 24 h; the cells were then transduced with Lenti/iNKT-sr39TK viruses for another 24 h following an established protocol^28^. The transduced HSCs were then collected and put into a 2-Stage *in vitro* HSC-iNKT culture.

At Stage 1, gene-engineered HSCs were differentiated into iNKT cells in an artificial thymic organoid (ATO) culture over 8 weeks. ATO was generated following a previously established protocol, with certain modifications^29^^,^^30^. Briefly, MS5-DLL4 cells were harvested by trypsinization and resuspended in serum free ATO culture medium (‘‘RB27’’) composed of RPMI 1640 (Corning), 4% B27 supplement (ThermoFisher Scientific), 30 mM L-ascorbic acid 2-phosphate sesquimagnesium salt hydrate (Sigma-Aldrich) reconstituted in PBS, 1% penicillin/streptomycin (Gemini Bio-Products), 1% Glutamax (ThermoFisher Scientific), 5 ng/ml rhFLT3L and 5 ng/ml rhIL-7 (Peprotech). RB27 was made fresh weekly. 1.5-6 × 10^5^ MS5-DLL4 cells were combined with 0.3-10 × 10^4^ transduced HSCs per ATO in 1.5 mL Eppendorf tubes (up to 12 ATOs per tube) and centrifuged at 300 g for 5 min at 4°C in a swinging bucket centrifuge. Supernatants were carefully removed, and the cell pellet was resuspended in 6 mL RB27 per ATO and mixed by brief vortexing. ATOs were plated on a 0.4 mm Millicell transwell insert (EMD Millipore; Cat. PICM0RG50) placed in a 6-well plate containing 1 mL RB27 per well. Medium was changed completely every 3-4 days by aspiration from around the cell insert followed by replacement with 1 mL fresh RB27/cytokines. ATO cells were harvested by adding FACS buffer (PBS/0.5% bovine serum album/2mM EDTA) to each well and briefly disaggregating the ATO by pipetting with a 1 mL ‘‘P1000’’ pipet, followed by passage through a 50 mm nylon strainer.

At Stage 2, isolated ATO cells comprising ^Allo^HSC-iNKT cells were expanded with αGC-loaded PBMCs (αGC-PBMCs). αGC-PBMCs were prepared by incubating 10^7^-10^8^ PBMCs in 5 mL C10 medium containing 5 μg/ml αGC for 1 h, followed by irradiation at 6,000 rads. ATO cells were mixed with irradiated αGC-PBMCs at ratio 1:1, followed by culturing in C10 medium supplemented with human IL-7 (10 ng/ml) and IL-15 (10 ng/ml) for 2 weeks; cell cultures were split and fresh media/cytokines were added if needed. The Stage 2 expansion culture could be extended to 3 weeks by adding additional αGC-PBMCs at ratio 1:1 at the end of week 2. At the end of Stage 2 culture, the resulting ^Allo^HSC-iNKT cell products were collected and cryopreserved for future use.

#### *In Vitro* Generation of BCMA CAR-Engineered ^Allo^HSC-iNKT (^Allo^BCAR-iNKT) Cells

^Allo^HSC-iNKT cells were generated in the 2-Stage HSC-iNKT culture as described above, followed by an additional Stage 3 CAR engineering culture. At one week into Stage 2 culture, ^Allo^HSC-iNKT cells were collected and stimulated with CD3/CD28 T-activator beads (ThermoFisher Scientific) in the presence of recombinant human IL-15 (10 ng/ml) and human IL-7 (10 ng/ml) for two days; the cells were then spin-infected with frozen-thawed Retro/BCAR-tEGFR retroviral supernatants supplemented with polybrene (10 μg/ml, Sigma-Aldrich) at 660 g at 30°C for 90 min. Retronectin (Takara) could be pre-coated on plate to increase transduction efficiency. After transduction, the resulting ^Allo^BCAR-iNKT cells were expanded for another 1-2 weeks in C10 medium supplemented with recombinant human IL-15 (10 ng/ml) and IL-7 (10 ng/ml), and then were collected and cryopreserved for future use.

#### *In Vitro* Generation of HLA-Ablated Universal HSC-iNKT (^U^HSC-iNKT) Cells

^U^HSC-iNKT cells were generated following the protocol of generating ^Allo^HSC-iNKT cells, with one additional gene-editing step. CD34^+^ HSCs were revived in HSC-culture medium on day 1, transduced with Lenti/iNKT-sr39TK viruses on day 2, and then were electroporated with a CRISPR-Cas9/B2M-CIITA-gRNAs complex on day 3, followed by entering the 2-Stage HSC-iNKT culture to generate ^U^HSC-iNKT cells. A small portion of engineered CD34^+^ HSCs were cultured in HSC-culture medium for 5 days, and flow cytometry was performed to evaluate the genome-editing efficiencies.

^U^BCAR-iNKT cells were generated following the protocol of generating ^Allo^BCAR-iNKT cells, with the same additional gene-editing step. CD34^+^ HSCs were revived in HSC-culture medium on day 1, transduced with Lenti/iNKT-sr39TK viruses on day 2, and then were electroporated with a CRISPR-Cas9/B2M-CIITA-gRNAs complex on day 3, followed by entering the 3-Stage BCAR-iNKT culture to generate ^U^BCAR-iNKT cells.

For electroporation, 2 × 10^5^ HSCs per condition were pelleted at 90 x g for 10 min at room temperature (RT), resuspended in 20 μL P3 solution (Lonza), mixed with pre-aliquoted B2M and CIITA gRNAs (1 μL of each gRNA at 100 μM) and Cas9 (4 μL at 6.5 mg/ml), and pulsed once at 250 V for 5 ms in an Amaxa 4D Nucleofector X Unit (Lonza). After electroporation, HSCs were rested at RT for 10 min, and then transferred to a 24-well tissue culture treated plate overnight before entering the 2-Stage HSC-iNKT or 3-Stage BCAR-iNKT culture.

If necessary, the resulting ^U^HSC-iNKT or ^U^BCAR-iNKT cell products collected at the end of the *in vitro* culture could be purified using Magnetic-Activate Cell Sorting (MACS) via B2M (Santa Cruz Biotechnology) and HLA-II magnetic beads labeling (Miltenyi Biotec), to enrich the HLA-I/II double negative cells in the final ^U^HSC-iNKT or ^U^BCAR-iNKT cell products.

#### Generation of PBMC-Derived Conventional αβT, iNKT, γδT, and NK Cells

Healthy donor PBMCs were obtained from the UCLA/CFAR Virology Core Laboratory, and were used to generate the PBMC-Tc, PBMC-iNKT, PBMC-γδT, and PBMC-NK cells.

To generate PBMC-Tc cells, PBMCs were stimulated with CD3/CD28 T-activator beads (ThermoFisher Scientific) and cultured in C10 medium supplemented with human IL-2 (20 ng/mL) for 2-3 weeks, following the manufacturer’s instructions.

To generate PBMC-iNKT cells, PBMCs were MACS-sorted via anti-iNKT microbeads (Miltenyi Biotech) labeling to enrich iNKT cells, which were then stimulated with donor-matched irradiated αGC-PBMCs at the ratio of 1:1, and cultured in C10 medium supplemented with human IL-7 (10 ng/ml) and IL-15 (10 ng/ml) for 2-3 weeks. If needed, the resulting PBMC-iNKT cells could be further purified using Fluorescence-Activated Cell Sorting (FACS) via human iNKT TCR antibody (Clone 6B11; BD Biosciences) staining.

To generate PBMC-γδT cells, PBMCs were stimulated with Zoledronate (5 μM; Sigma-Aldrich) and cultured in C10 medium supplemented with human IL-2 (20 ng/ml) for 2 weeks. If needed, the resulting PBMC-γδT cells could be further purified using FACS via human TCR Vδ2 antibody (Clone B6; Biolegend) staining or via MACS using a human TCRγ/δ T Cell Isolation Kit (Miltenyi Biotech).

To generate PBMC-NK cells, PBMCs were FACS-sorted via human CD56 antibody (Clone HCD56; Biolegend) labeling or MACS-sorted using a human NK Cell Isolation Kit (Miltenyi Biotech).

#### Generation of BCMA CAR-Engineered PBMC T (BCAR-T) cells

Healthy donor PBMCs were stimulated with CD3/CD28 T-activator beads (ThermoFisher Scientific) in the presence of recombinant human IL-2 (30 ng/ml), following the manufacturer’s instructions. On day 2, cells were spin-infected with frozen-thawed Retro/BCAR-tEGFR retroviral supernatants supplemented with polybrene (10 μg/ml, Sigma-Aldrich) at 660 g at 30°C for 90 min. Retronectin (Takara) could be pre-coated on plate to increase transduction efficiency. The resulting BCAR-T cells were expanded for another 7-10 days, and then were cryopreserved for future use.

#### Single Cell TCR Sequencing

^Allo^HSC-iNKT (6B11^+^TCRαβ^+^), PBMC-iNKT (6B11^+^TCRαβ^+^), and PBMC-Tc (6B11^-^TCRαβ^+^) cells were sorted using a FACSAria II flow cytometer. Sorted cells were immediately delivered to the UCLA TCGB (Technology Center for Genomics and Bioinformatics) Core to perform single cell TCR sequencing using a 10X Genomics Chromium™ Controller Single Cell Sequencing System (10X Genomics), following the manufacturer’s instructions and the TCGB Core’s standard protocol. Libraries were constructed using an Illumina TruSeq RNA Sample Prep Kit (Cat#FC-122-1001) and sequenced with 150 bp paired end reads (5,000 reads/cell) on an Illumina NovaSeq. The reads were mapped to the human T cell receptor reference genome (hg38) using Cell Ranger VDJ. The frequencies of the α or β chain recombination were plotted.

#### Deep RNA Sequencing (Deep RNaseq) and Data Analysis

A total of 25 cell samples were analyzed, as described in the following table:

**Table:** 

Sample name	Number of replicates (from different donors)	FACS sorting markers	Description
^Allo^HSC-iNKT (from PBSC)	3	6B11^+^TCRαβ^+^	^Allo^HSC-iNKT cells derived from G-CSF mobilized peripheral blood CD34^+^ HSCs
^Allo^HSC-iNKT (from CB)	3	6B11^+^TCRαβ^+^	^Allo^HSC-iNKT cells derived from cord blood CD34^+^ HSCs
PBMC-iNKT (CD4^-^)	3	6B11^+^TCRαβ^+^CD4^-^	PBMC-iNKT cells derived from healthy donor PBMCs (CD4^-^ cells were analyzed)
PBMC-αβTc (CD4^-^)	8	6B11^-^TCRαβ^+^CD4^-^	PBMC-αβTc cells derived from healthy donor PBMCs (CD4^-^ cells were analyzed)
PBMC-NK	2	CD56^+^TCRαβ^-^	PBMC-NK cells derived from healthy donor PBMCs
PBMC-γδT	6	TCRγδ^+^TCRαβ^-^	PBMC-γδT cells derived from healthy donor PBMCs

Both ^Allo^HSC-iNKT and PBMC-iNKT cells were activated *in vitro* with αGC, PBMC-αβTc cells were stimulated with CD3/CD28 T-activator beads, and PBMC-γδT cells were stimulated with Zoledronate. Cell samples were sorted using a FACSAria II flow cytometer. Total RNAs were isolated from each cell sample using an miRNeasy Mini Kit (QIAGEN) and delivered to the UCLA TCGB Core to perform Deep RNA sequencing using an Illumina HiSeq3000, following the manufacturer’s instructions and the TCGB Core’s standard protocol. cDNAs were synthesized using an iScript cDNA Synthesis Kit (1708890, BioRad). Libraries were constructed using an Illumina TruSeq Stranded Total RNA Sample Prep kit and sequenced with 50 bp single end reads (20 M reads/sample) on an Illumina HiSeq3000. The reads were mapped with STAR 2.5.3a to the human genome (hg38). The counts for each gene were obtained using–quantMode GeneCounts in STAR commands, and the other parameters during alignment were set to default. Data quality was checked using Illumina’s proprietary software. Sequencing depth normalized counts were obtained from the differential expression analysis and were used for principal component analysis.

#### ^Allo^HSC-iNKT Cell Phenotype and Functional Study

^Allo^HSC-iNKT cells and their derivatives (i.e., ^Allo^BCAR-iNKT, ^U^HSC-iNKT, and ^U^BCAR-iNKT cells) were analyzed in comparison with PBMC-Tc, PBMC-NK, PBMC-iNKT, or/and BCAR-T cells. Phenotype of these cells was studied using flow cytometry, by analyzing cell surface markers including co-receptors (i.e., CD4 and CD8), NK cell receptors (i.e., CD161, NKG2D, DNAM-1, and KIR), memory T cell markers (i.e., CD45RO), and inflammatory tissue/tumor homing markers (i.e., CCR4, CCR5, and CXCR3). Capacity of these cells to produce cytokines (i.e., IFN-γ, TNF-α, and IL-2) and cytotoxic molecules (i.e., perforin and granzyme B) were studied using flow cytometry via intracellular staining.

Response of ^Allo^HSC-iNKT cells to antigen stimulation was studied by culturing ^Allo^HSC-iNKT cells *in vitro* in C10 medium for 7 days, in the presence or absence of αGC (100 ng/ml). Proliferation of ^Allo^HSC-iNKT cells was measured by cell counting and flow cytometry (identified as 6B11^+^TCRαβ^+^) over time. Cytokine production was assessed by ELISA analysis of cell culture supernatants collected on day 7 (for human IFN-γ. TNF-α, IL-2, IL-4, and IL-17).

#### *In Vitro* Tumor Cell Killing Assay

Tumor cells (1 × 10^4^ cells per well) were co-cultured with effector cells (at ratios indicated in figure legends) in Corning 96-well clear bottom black plates for 8-24 h, in C10 medium with or without the addition of αGC (100 ng/ml). At the end of culture, live tumor cells were quantified by adding D-luciferin (150 μg/ml; Caliper Life Science) to cell cultures and reading out luciferase activities using an Infinite M1000 microplate reader (Tecan).

In some experiments, 10 μg/ml of LEAF™ purified anti-human NKG2D (Clone 1D11, Biolegend), anti-human DNAM-1 antibody (Clone 11A8, Biolegend), or LEAF™ purified mouse lgG2bk isotype control antibody (Clone MG2B-57, Biolegend) was added to co-cultures, to study NK activating receptor-mediated tumor cell killing mechanism.

All the PBMC-derived cells and HSC-derived iNKT cells were cryopreserved before use. The cells for comparison (e.g., ^Allo^HSC-iNKT and PBMC-NK cells in Figure 3) were cryopreserved and thawed for testing side-by-side using Mr. Frosty Freezing Container (Thermo Scientific Nalgene) following the manufacture’s instructions.

#### *In Vitro* Mixed Lymphocyte Reaction (MLR) Assay: Studying Graft-Versus-Host (GvH) Response

PBMCs of multiple healthy donors were irradiated at 2,500 rads and used as stimulators, to study the GvH response of ^Allo^HSC-iNKT cells and their derivatives (i.e., ^Allo^BCAR-iNKT and ^U^BCAR-iNKT cells) as responders. PBMC-Tc or BCAR-T cells were included as responder controls. Stimulators (5 × 10^5^ cells/well) and responders (2 × 10^4^ cells/well) were co-cultured in 96-well round bottom plates in C10 medium for 4 days; the cell culture supernatants were then collected to measure IFN-γ production using ELISA.

#### *In Vitro* MLR Assay: Studying Host-Versus-Graft (HvG) Response

PBMCs of multiple healthy donors were used as responders, to study the HvG response of ^Allo^HSC-iNKT cells and their derivatives (i.e., ^Allo^BCAR-iNKT and ^U^BCAR-iNKT cells) as stimulators (irradiated at 2,500 rads). PBMC-Tc, PBMC-iNKT or BCAR-T cells were included as stimulator controls. Stimulators (5 × 10^5^ cells/well) and responders (2 × 10^4^ cells/well) were co-cultured in 96-well round bottom plates in C10 medium for 4 days; the cell culture supernatants were then collected to measure IFN-γ production using ELISA.

#### *In Vitro* MLR Assay: Studying NK Cell-Mediated Allorejection

PBMC-NK cells isolated from PBMCs of multiple healthy donors were used to study the NK cell-mediated allorejection of ^Allo^HSC-iNKT cells and their derivatives (i.e., ^Allo^BCAR-iNKT and ^U^BCAR-iNKT cells). Allogeneic PBMC-Tc or PBMC-iNKT cells were included as controls. PBMC-NK cells (2 × 10^4^ cells/well) and the corresponding allogeneic cells (2 × 10^4^ cells/well) were co-cultured in 96-well round bottom plates in C10 medium for 24 h; the cell cultures were then collected to quantify live cells using flow cytometry.

#### Bioluminescence Live Animal Imaging (BLI)

BLI was performed using an IVIS 100 imaging system (Xenogen/PerkinElmer) or a Spectral Advanced Molecular Imaging (AMI) HTX imaging system (Spectral instrument Imaging). Live animal imaging was acquired 5 min after intraperitoneal (i.p.) injection of D-Luciferin (1 mg per mouse). Imaging results were analyzed using a Living Imaging 2.50 software (Xenogen/ PerkinElmer) or an AURA imaging software (Spectral Instrument Imaging).

#### ^Allo^HSC-iNKT Cell *In Vivo* Antitumor Efficacy Study: A375 Human Melanoma Xenograft NSG Mouse Model

NSG mice were pre-conditioned with 100 rads of total body irradiation (day −1), followed by subcutaneous inoculation with 1 × 10^6^ A375-FG cells (day 0). On day 3, the tumor-bearing experimental mice received intravenous (i.v.) injection of vehicle (PBS), 1.2 × 10^7 Allo^HSC-iNKT cells, or 1.2 × 10^7^ PBMC-NK cells. Over time, tumor loads were monitored by measuring total body luminescence using BLI and by measuring tumor size using a Fisherbrand™ Traceable™ digital caliper (Thermo Fisher Scientific). The tumor size was calculated as W x L mm^2^. At around week 3, mice were terminated, and solid tumors were retrieved and weighted using a PA84 precision balance (Ohaus).

#### ^Allo^BCAR-iNKT Cell *In Vivo* Antitumor Efficacy Study: MM.1S Human MM Xenograft NSG Mouse Model

NSG mice were pre-conditioned with 175 rads of total body irradiation (day −1), followed by intravenous inoculation with 1 × 10^6^ MM-CD1d-FGFP cells (day 0). To study antitumor efficacy under low tumor load condition, on day 3, the tumor-bearing experimental mice received i.v. injection of vehicle (PBS), ^Allo^BCAR-iNKT cells (7 × 10^6^ CAR^+^ cells), or conventional BCAR-T cells (7 × 10^6^ CAR^+^ cells). To study antitumor efficacy under high tumor load condition, on day 15, the tumor-bearing experimental mice received i.v. injection of vehicle (PBS), ^Allo^BCAR-iNKT cells (5 × 10^6^ CAR^+^ cells), or conventional BCAR-T cells (5 × 10^6^ CAR^+^ cells). Over time, experimental mice were monitored for survival, and their tumor loads were measured using BLI.

#### ^U^BCAR-iNKT Cell *In Vivo* Antitumor Efficacy Study: MM.1S Human MM Xenograft NSG Mouse Model

NSG mice were pre-conditioned with 175 rads of total body irradiation (day −1), followed by intravenous inoculation with 5 × 10^5^ MM-CD1d-FGFP cells (day 0). On day 3 the tumor-bearing experimental mice received i.v. injection of vehicle (PBS), ^Allo^BCAR-iNKT cells (3 × 10^6^ CAR^+^ cells), or conventional BCAR-T cells (3 × 10^6^ CAR^+^ cells). Over time, experimental mice were monitored for survival, and their tumor loads were measured using BLI.

#### Ganciclovir (GCV) *In Vitro* and *In Vivo* Killing Assay

For GCV *in vitro* killing assay, ^Allo^HSC-iNKT cells were cultured in C10 medium in the presence of titrated amount of GCV (0-50 μM) for 4 days; live ^Allo^HSC-iNKT cells were then counted using a hematocytometer (VWR) via Trypan Blue staining (Fisher Scientific).

GCV *in vivo* killing assay was performed using an NSG xenograft mouse model. NSG mice received i.v. injection of 1 × 10^7 Allo^HSC-iNKT cells on day 0, followed by i.p. injection of GCV for 5 consecutive days (50 mg/kg per injection per day). On day 5, mice were terminated. Multiple tissues (i.e., spleen, liver, and lung) were collected and processed for flow cytometry analysis to detect tissue-infiltrating ^Allo^HSC-iNKT cells (identified as iNKT TCR^+^CD45^+^), following established protocols.^28^

#### Histological Analysis

Tissues (i.e., heart, liver, kidney, lung, and spleen) were collected from the experimental mice, fixed in 10% Neutral Buffered Formalin for up to 36 h, then embedded in paraffin for sectioning (5 μm thickness). Tissue sections were prepared and stained with Hematoxylin and Eosin (H&E) by the UCLA Translational Pathology Core Laboratory, following the Core’s standard protocols. Stained sections were imaged using an Olympus BX51 upright microscope equipped with an Optronics Macrofire CCD camera (AU Optronics) at 20 x and 40 x magnifications. The images were analyzed using Optronics PictureFrame software (AU Optronics).

#### Statistical Analysis

GraphPad Prism 6 (Graphpad Software) was used for statistical data analysis. Student’s two-tailed t test was used for pairwise comparisons. Ordinary 1-way ANOVA followed by Tukey’s or Dunnett’s multiple comparisons test was used for multiple comparisons. Log rank (Mantel-Cox) test adjusted for multiple comparisons was used for Meier survival curves analysis. Data are presented as the mean ± SEM, unless otherwise indicated. In all figures and figure legends, “n” represents the number of samples or animals utilized in the indicated experiments. A P value of less than 0.05 was considered significant. ns, not significant; ∗p < 0.05; ∗∗p < 0.01; ∗∗∗p < 0.001; ∗∗∗∗p < 0.0001.

## Data Availability

• The genomics data generated during this study are available from the public repository Gene Expression Omnibus Database: GSE164425, GSE164500. • All data reported in this manuscript are available from the Lead Contact without restriction • No custom computer code was reported in this manuscript. • Any additional information required to reanalyze the data reported in this work paper is available from the Lead Contact upon request.

## Supplemental information

Document S1. Figures S1–S7

Document S2. Article plus supplemental information
