# Supplementary material for: Development of allogeneic HSC-engineered iNKT cells for off-the-shelf cancer immunotherapy
Source: Cell Rep Med. 2021 Nov 16;2(11):100449. doi: 10.1016/j.xcrm.2021.100449 (PMC8607011; doi:10.1016/j.xcrm.2021.100449)
Supplement: Document S1. Figures S1–S7 [file mmc1.pdf]

Cell Reports Medicine, Volume *2*

Supplemental information

Development of allogeneic HSC-engineered iNKT cells for off-the-shelf cancer immunotherapy

Yan-Ruide Li, Yang Zhou, Yu Jeong Kim, Yanni Zhu, Feiyang Ma, Jiaji Yu, Yu-Chen Wang, Xianhui Chen, Zhe Li, Samuel Zeng, Xi Wang, Derek Lee, Josh Ku, Tasha Tsao, Christian Hardoy, Jie Huang, Donghui Cheng, Amélie Montel-Hagen, Christopher S. Seet, Gay M. Crooks, Sarah M. Larson, Joshua P. Sasine, Xiaoyan Wang, Matteo Pellegrini, Antoni Ribas, Donald B. Kohn, Owen Witte, Pin Wang, and Lili Yang

Supplemental Information

Development of Allogeneic HSC-Engineered iNKT cells for Off-the-Shelf Cancer Immunotherapy

Yan-Ruide Li, Yang Zhou Yu Jeong Kim, Yanni Zhu, Feiyang Ma, Jiaji Yu, Yu-Chen Wang, Xianhui Chen, Zhe Li, Samuel Zeng, Xi Wang, Derek Lee, Josh Ku, Tasha Tsao, Christian Hardoy, Jie Huang, Donghui Cheng, Amélie Montel-Hagen, Christopher Seet, Gay M. Crooks, Sarah M. Larson, Joshua P. Sasine, Matteo Pellegrini, Antoni Ribas, Donald B. Kohn, Owen Witte, Pin Wang, and Lili Yang

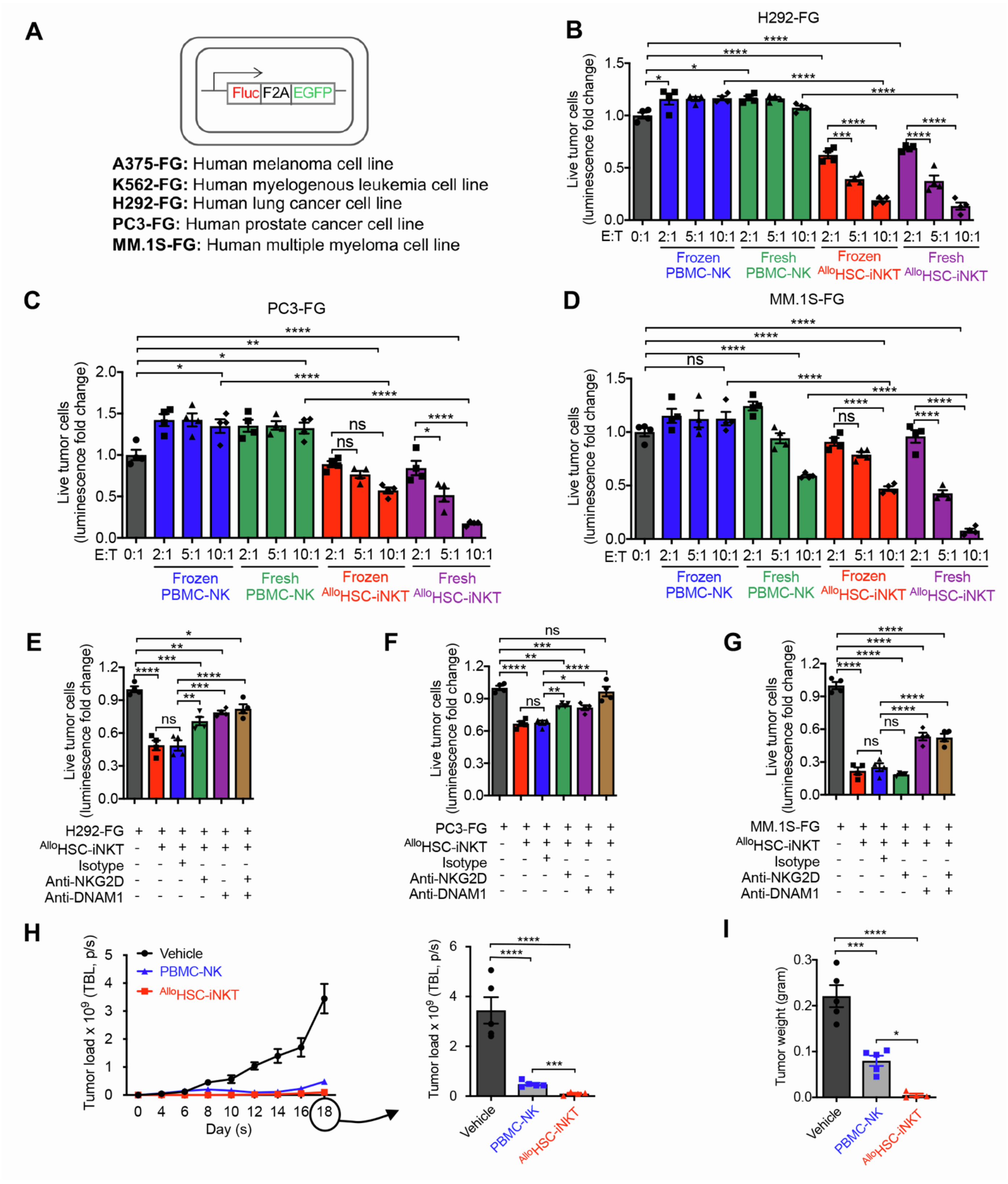

Figure S1. Tumor Targeting of ^{Allo}HSC-iNKT Cells Through Intrinsic NK Function; Related to Figure 3.

(A) Schematics showing the engineered A375-FG, K562-FG, H292-FG, PC3-FG, and MM.1S-FG cell lines. Fluc, firefly luciferase; EGFP, enhanced green fluorescent protein; F2A, foot-and-mouth disease virus 2A self-cleavage sequence.
(B-D) *In vitro* direct killing of human tumor cells by ^{Allo}HSC-iNKT cells. PBMC-NK cells were included as a control. Both fresh and frozen-thawed cells were studied. Tumor cell killing was analyzed at 24-hours post co-culture. Tumor killing data of H292-FG human lung cancer cells (B), PC3-FG human prostate cancer cells (C), and MM.1S-FG human multiple myeloma cells (D) were presented. N = 4. Related to main Figures 3C-3E.
(E-G) Tumor killing mechanisms of ^{Allo}HSC-iNKT cells. NKG2D and DNAM-1 mediated pathways were studied. Tumor cell killing was analyzed at 24-hours post co-culture. Tumor killing data of H292-FG (tumor:iNKT ratio 1:2), PC3-FG (tumor:iNKT ratio 1:10), and MM.1S-FG (tumor:iNKT ratio 1:15) were presented. N = 4. Related to main Figures 3F-3H.
(H-I) *In vivo* antitumor efficacy of ^{Allo}HSC-iNKT cells in an A375-FG human melanoma xenograft NSG mouse model. (H) BLI measurements of tumor loads over time (n = 4 or 5). (I) Tumor weight at the terminal harvest on day 18 (n = 4 or 5). Related to main Figures 3I-3K.
Representative of 3 experiments. Data are presented as the mean ± SEM. ns, not significant, *P < 0.05, **P < 0.01, ***P < 0.001, ****P < 0.0001, by 1-way ANOVA (B-G, I) or by Student's *t* test (H).

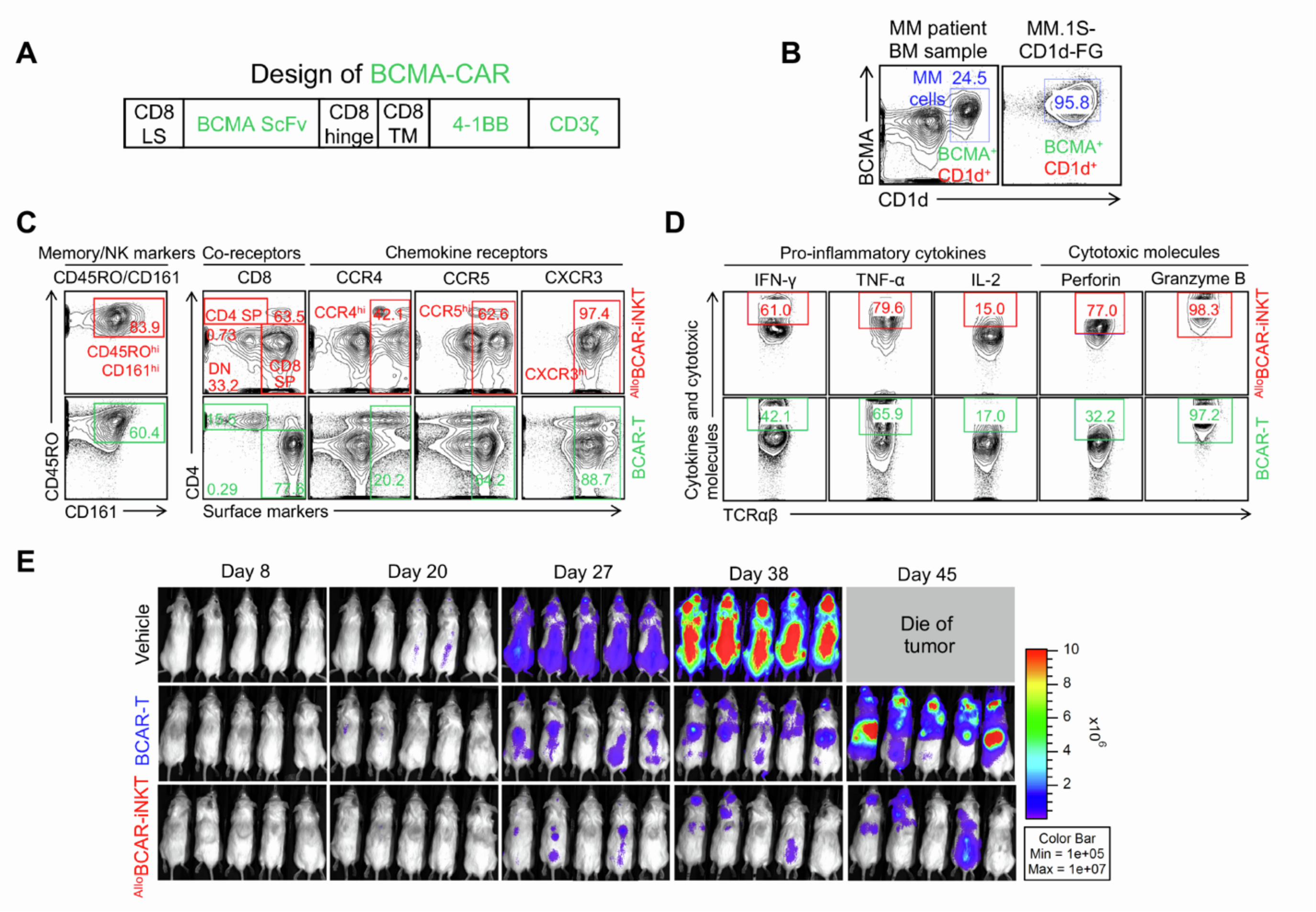

Figure S2. Tumor Targeting of ^{Allo}HSC-iNKT Cells Through Engineered Chimeric Antigen Receptors; Related to Figure 4.

(A) Schematics showing the design of BCMA-CAR. LS, leader sequence; ScFv, single-chain variable fragment; TM, transmembrane domain.

(B) FACS analyses of BCMA and CD1d expression on MM.1S-CD1d-FG cells. A representative MM patient primary bone marrow (BM) sample was included as a control.

(C-D) FACS characterization of ^{Allo}BCAR-iNKT cells. (C) Surface marker expression. (D) Intracellular cytokine and cytotoxic molecule production. BCAR-T cells were included as a control.

(E) *In vivo* antitumor efficacy of ^{Allo}BCAR-iNKT cells in an MM.1S-CD1d-FG human multiple myeloma xenograft NSG mouse model. BLI images were presented showing tumor loads in experimental mice over time. Related to main Figures 4K-4N.

Representative of 2 (E) and 3 (A-D) experiments.

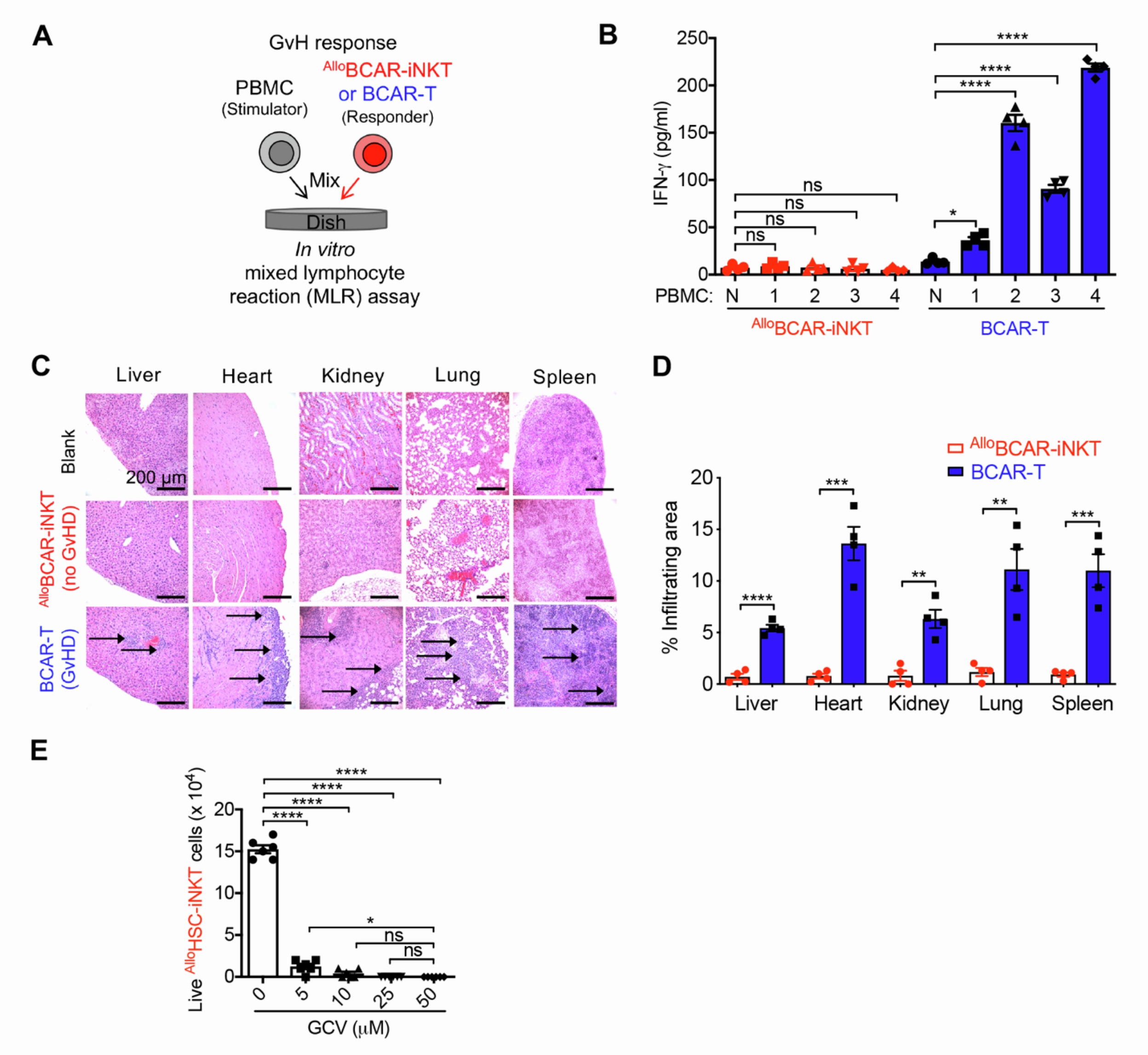

Figure S3. Safety study of ^{Allo}HSC-iNKT cells; Related to Figure 5.

(A-B) Studying the graft-verus-host (GvH) response of ^{Allo}BCAR-iNKT cells using an *in vitro* mixed lymphocyte reaction (MLR) assay. BCAR-T cells were included as a responder cell control. (A) Experimental design. PBMCs from 4 different healthy donors were used as stimulator cells. (B) ELISA analyses of IFN-γ production at day 4 (n = 4).

(C-D) Histology analysis of tissue sections collected from experimental mice as described in Figures 4G-4J. (C) H&E-stained tissue sections. Blank indicates tissue sections collected from NSG mice receiving no adoptive cell transfer. Arrows point to mononuclear cell infiltrates. Scale bar: 200 µm. (D) Quantification of C (n = 4).

(E) *In vitro* GCV killing assay. ^{Allo}HSC-iNKT cells were cultured *in vitro* in the presence of gradient concentrations of GCV for 4 days, followed by quantification of live cells via cell counting (n = 6).

Representative of 2 experiments. Data are presented as the mean ± SEM. ns, not significant, *P < 0.05, **P < 0.01, ***P < 0.001, ****P < 0.0001, by Student's *t* test (D) or by 1-way ANOVA (B and E).

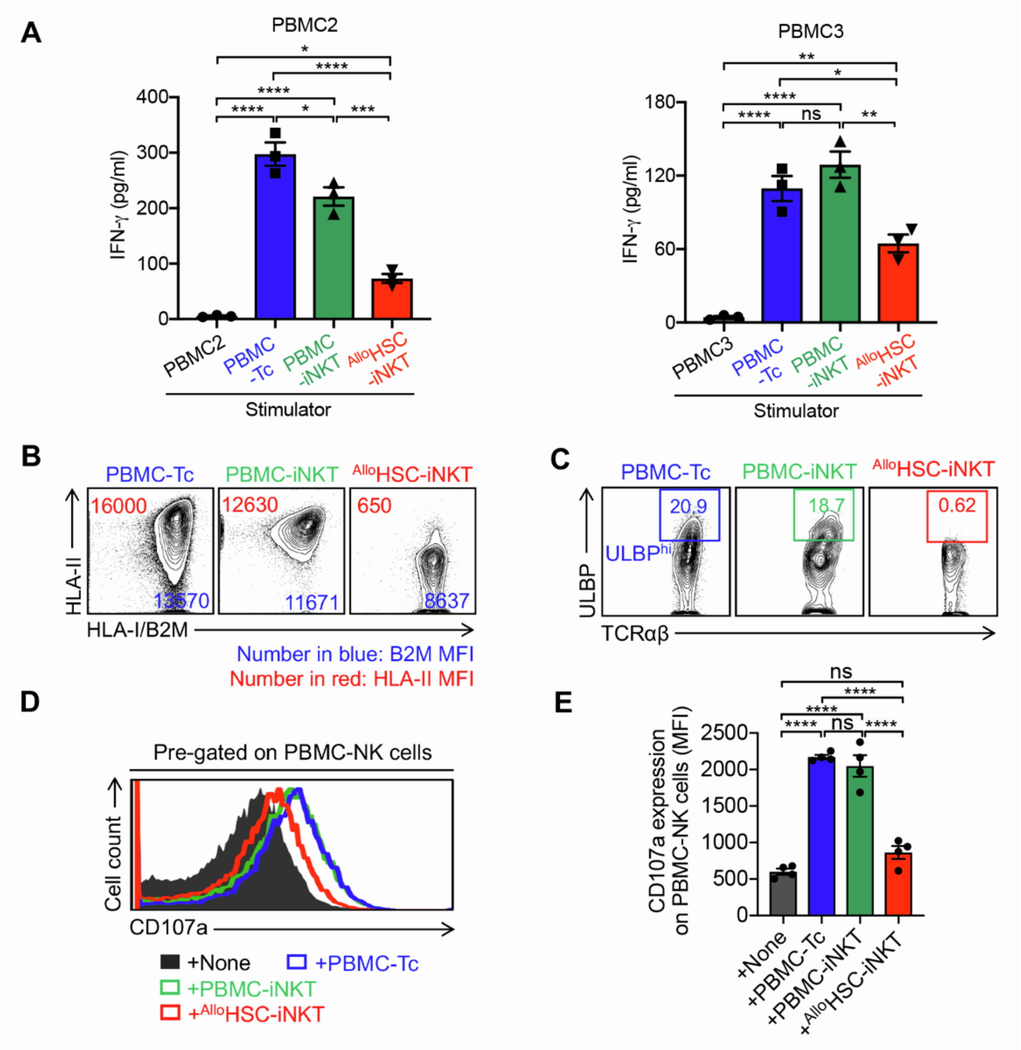

Figure S4. Immunogenicity Study of ^{Allo}HSC-iNKT Cells; Related to Figure 6.

(A) ELISA analyses of IFN-γ production at day 4 in an *in vitro* MLR assay (n = 3). Data of PBMC2 and PBMC3 responders were presented. Related to main Figures 6A and 6C.

(B) FACS analyses of HLA-I/II expression on PBMC-Tc, PBMC-iNKT, and ^{Allo}HSC-iNKT cells. Representative FACS plots were presented. Related to main Figure 6B.

(C) FACS analyses of ULBP expression on PBMC-Tc, PBMC-iNKT, and ^{Allo}HSC-iNKT cells. Representative FACS plots were presented. Related to main Figure 6F.

(D-E) Studying CD107a expressions on PBMC-NK cells using an *in vitro* MLR assay. PBMC-NK cells were co-cultured with ^{Allo}HSC-iNKT, PBMC-iNKT, and PBMC-Tc cells for 24 hours. CD107a antibody was added into the cell cultures and incubated for 2 hours prior to flow cytometry. (D) FACS analyses of CD107a expression on PBMC-NK cells. (E) Quantification of D (n = 4).

Representative of 3 experiments. Data are presented as the mean ± SEM. ns, not significant, *P < 0.05, **P < 0.01, ***P < 0.001, ****P < 0.0001, by one-way ANOVA.

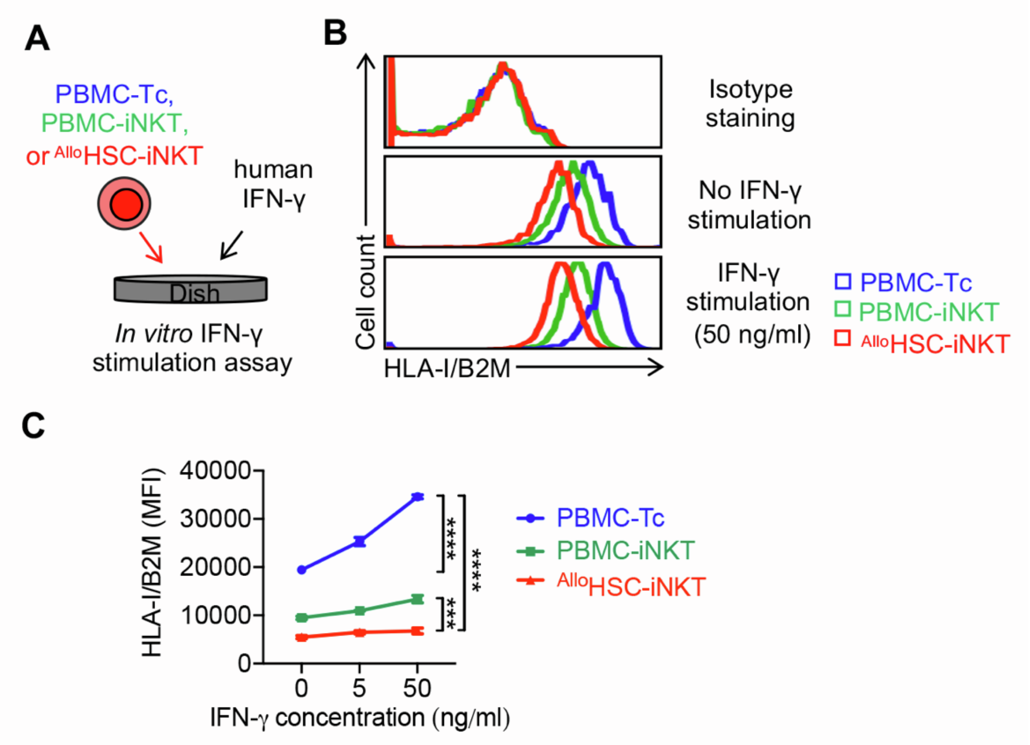

Figure S5. HLA-I Expression Study on ^{Allo}HSC-iNKT Cells Under IFN-γ Stimulation; Related to Figure 6.

(A) Experimental design. ^{Allo}HSC-iNKT cells were stimulated with a range of IFN-γ (0, 5, and 50 ng/ml) for 3 days. PBMC-iNKT and PBMC-Tc cells were included as controls.

(B) FACS analyses of HLA-I expression on the indicated cells.

(C) Quantification of HLA-I expression on the indicated cells stimulated with IFN-γ of indicated concentrations (n = 3).

Representative of 3 experiments. Data are presented as the mean ± SEM. *P < 0.05, ***P < 0.001, ****P < 0.0001, by 1-way ANOVA.

A

Set up \ MLR	MLR1	MLR2	MLR3	MLR4	MLR5
PBMC-NK	Donor 1	Donor 2	Donor 3	Donor 4	Donor 5
PBMC-Tc	Donor 6	Donor 6	Donor 7	Donor 7	Donor 8
AlloHSC-iNKT	Donor 9	Donor 10	Donor 9	Donor 7	Donor 8
PBMC-iNKT	Donor 11	Donor 11	Donor 11	Donor 12	Donor 12

Shown in main Figure 6I (MLR1)

PBMC-Tc and AlloHSC-iNKT from the same donor (MLR4, MLR5)

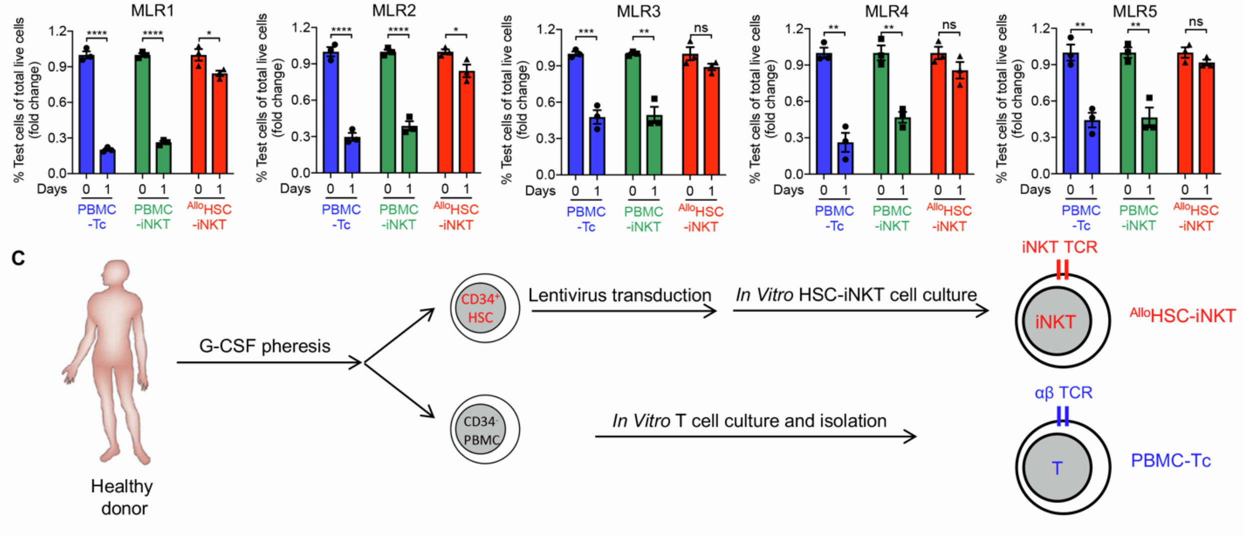

Figure S6. Studying NK cell-mediated allorejection of AlloHSC-iNKT cells; Related to Figure 6.
(A) Table showing the donor information of MLR assay. Five different sets of MLR assay were performed; three sets used PBMC-Tc and AlloHSC-iNKT cells from different donors, two sets used PBMC-Tc and AlloHSC-iNKT cells from the same donors. Data of MLR1 have been shown in Figure 6I.
(B) Quantification of the indicated test cells (n = 3).
(C) Diagram showing the experimental procedure to generate PBMC-Tc and AlloHSC-iNKT cells from the same donor.
Data are presented as the mean ± SEM. ns, not significant, *P < 0.05, **P < 0.01, ***P < 0.001, ****P < 0.0001, by Student's *t* test.

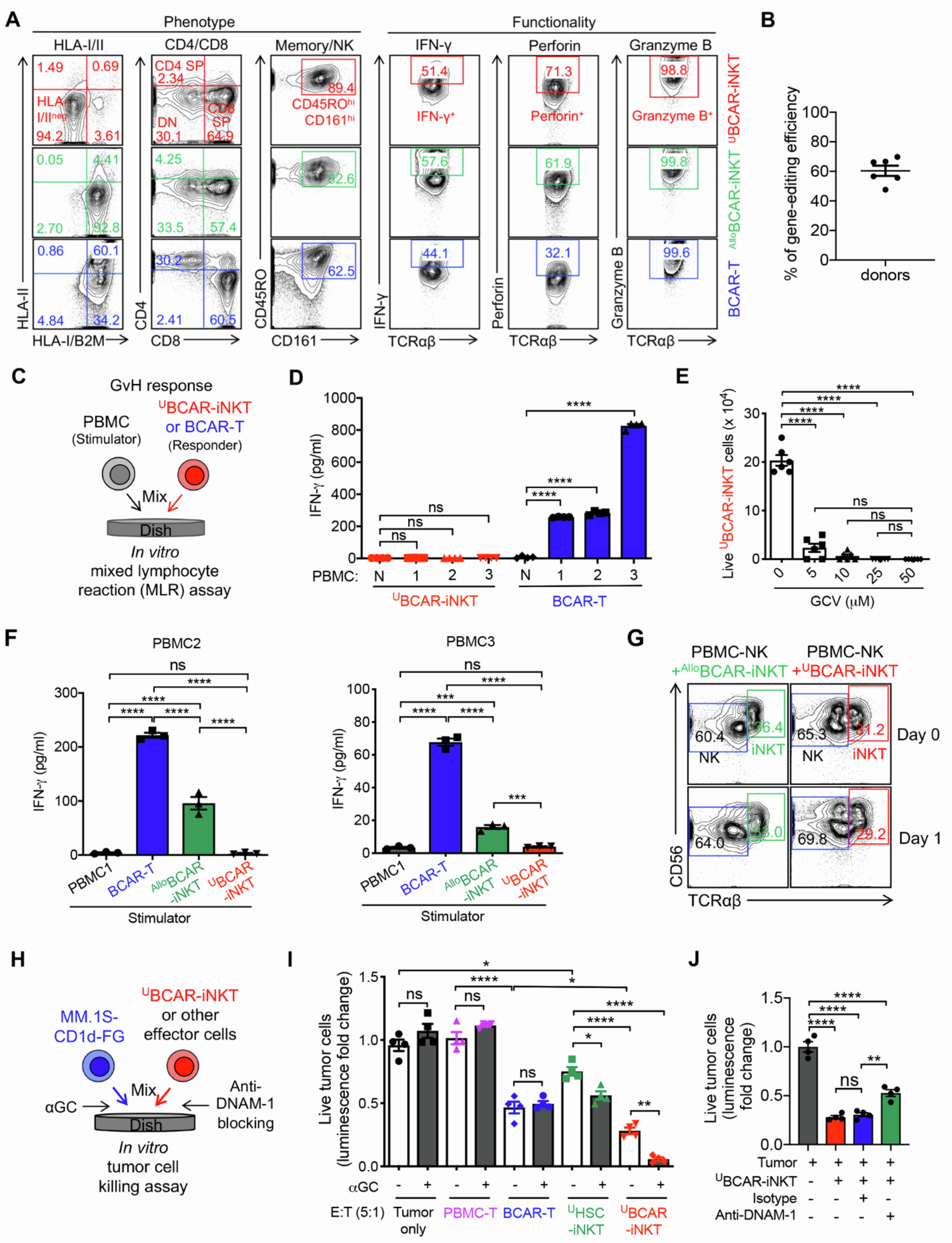

Figure S7. Development of HLA-Ablated Universal HSC-iNKT (UHSC-iNKT) Cells and Derivatives; Related to Figure 7.

(A) FACS analyses of surface marker expression, and Intracellular cytokine and cytotoxic molecule production by ^{U}BCAR-iNKT cells. ^{Allo}BCAR-iNKT and BCAR-T cells were included as controls.
(B) The genome editing efficiencies among multiple HSC donors.
(C-D) Studying the GvH response of ^{u}BCAR-iNKT cells using an *in vitro* MLR assay. BCAR-T cells were included as a responder cell control. (C) Experimental design. PBMCs from 3 different healthy donors were used as stimulator cells. (D) ELISA analyses of IFN-γ production at day 4 (n = 4).
(E) *In vitro* GCV killing assay. ^{U}BCAR-iNKT cells were cultured *in vitro* in the presence of gradient concentrations of GCV for 4 days, followed by quantification of live cells via cell counting (n = 6).
(F) Studying allogenic T cell response against ^{U}BCAR-iNKT cells using an *in vitro* MLR assay. ELISA analyses of IFN-γ production at day 4 were presented (n = 3). Related to main Figures 7E and 7F.
(G) Studying allogenic NK cell response against ^{U}BCAR-iNKT cells using an *in vitro* MLR assay. ^{Allo}BCAR-iNKT cells were included as a control. Representative FACS plots were presented, showing the quantification of the indicated cells at day 0 and day 1. Related to main Figures 7G and 7H.
(H-J) *In vitro* killing of MM.1S-CD1d-FG human multiple myeloma cells by ^{U}BCAR-iNKT cells. PBMC-T, BCAR-T, and ^{U}HSC-iNKT cells were included as effector cell controls. (H) Experimental design. (I) Tumor cell killing by the indicated effector cells with/out the addition of αGC (n = 4). (J) Tumor cell killing by ^{Allo}BCAR-iNKT cells with/out the blockade of DNAM-1 (n = 4). Tumor cell killing was analyzed at 8-hours post co-culture (effector:tumor ratio 5:1).
Representative of 3 experiments. Data are presented as the mean ± SEM. ns, not significant, *P < 0.05, **P < 0.01, ***P < 0.001, ****P < 0.0001, by 1-way ANOVA.
